# Disordered N‐termini enhance the thermostability of SGNH‐hydrolase family polyesterases

**DOI:** 10.1002/pro.70402

**Published:** 2025-12-22

**Authors:** F. Hafna Ahmed, Lygie Esquirol, Santana Royan, Mitchell M. Birgan, Nigel G. French, Sophia Newton, Alessandro T. Caputo, Colin Scott

**Affiliations:** ^1^ Environment, CSIRO Canberra Australian Capital Territory Australia; ^2^ Advanced Engineering Biology Future Science Platform, CSIRO Canberra Australian Capital Territory Australia; ^3^ Manufacturing, CSIRO Clayton Victoria Australia; ^4^ ARC Centre of Excellence in Synthetic Biology Canberra ACT Australia; ^5^ School of Molecular Sciences, The University of Western Australia Perth Western Australia Australia

**Keywords:** esterase, intrinsically disordered regions, plastic degrading enzymes, polyesterase, protein thermostability, SGNH hydrolase

## Abstract

Polyesters are widely used plastics that persist in the environment due to their resistance to degradation. Microbial polyesterases potentially offer recycling and remediation solutions. However, most polyesterases lack the thermostability and catalytic efficiency required for practical application. Here, we have identified and characterized thermostable bacterial polyesterases from an undercharacterized subfamily of SGNH‐hydrolases (related to PpEST from *Pseudomonas oleovorans*) and uncovered a previously unreported thermal stabilization mechanism mediated by conformationally flexible N‐terminal regions with features of intrinsically disordered regions. Biochemical assays, structural analysis, small angle X‐ray scattering, X‐ray crystallography, and molecular dynamics simulations suggest that these flexible N‐terminal regions enhance thermal resilience without affecting the catalytic rate or oligomerization in some homologs, while they promote oligomerization and reduce *k*_cat_ in others. These findings suggest that flexible terminal regions can act as modular stabilizing elements through diverse mechanisms. Our work provides mechanistic insight into an unusual route to protein thermostability, expanding strategies for enzyme engineering, and contributing to the development of robust biocatalysts for polyester degradation.

## 1 INTRODUCTION

Polyesters are widely used plastics with diverse applications in food packaging, textiles, and consumer goods. Their durability, low reactivity, and low production cost have driven their global adoption, but have also inadvertently contributed to widespread environmental pollution (Moharir & Kumar, 2019; Worm et al., 2017). Particularly, the prolific use of single‐use plastic items combined with inadequate waste disposal and management practices has caused increased polyester and other plastic waste in natural environments (Moharir & Kumar, 2019; Rochman, 2018; Worm et al., 2017). Aromatic polyesters, such as polyethylene terephthalate (PET), dominate the polyester market due to their high durability and crystallinity, which also limits their biodegradability (Mueller, 2006). While polyesters are more susceptible to hydrolysis than polyolefins due to their ester linkages (Shi et al., 2009), degradation in the environment can still take centuries (Moharir & Kumar, 2019).

Compared to petroleum‐derived polyesters such as PET, bioplastics that are fully or partially derived from renewable biological sources or specifically engineered to biodegrade more readily are becoming increasingly popular (Rosenboom et al., 2022; Yu & Flury, 2024). These polyesters are either aliphatic polymers or aliphatic‐aromatic co‐polymers, such as polybutylene succinate co‐adipate (PBSA), polybutylene adipate co‐terephthalate (PBAT), and polylactic acid, which degrade more readily under specific natural or controlled composting conditions (Tserki et al., 2006; Witt et al., 1999).

The rate of polyester biodegradation in natural environments depends on the local physical, chemical, and microbial conditions (Kijchavengkul et al., 2010; Puchalski et al., 2018). Several microbes degrade polyesters using serine hydrolases evolved from cutinases, esterases, and lipases that hydrolyze natural aryl and acyl ester substrates such as esters of ω‐hydroxy fatty acids, soluble carboxyl esters, and lactones (Kaushal et al., 2021; Mohanan et al., 2020; Mueller, 2006; Wang et al., 2010). However, the activity of these naturally evolved enzymes toward synthetic polyesters is usually inefficient for biotechnology and engineering biology applications that tackle plastics waste (Wei & Zimmermann, 2017). Despite this limitation, the enzymatic depolymerization of polyesters, such as PET, to chemically regenerate and reuse the constituent monomers in a circular economy is a rapidly growing industry (Rosenboom et al., 2022; Wei & Zimmermann, 2017). Additionally, embedding enzymes into plastics during production has emerged as a novel approach to accelerate biodegradation post‐disposal (DelRe et al., 2021; Greene et al., 2021).

Alongside high catalytic efficiency, enzymes used for the industrial recycling and manufacturing of plastics need to have high thermal stability. Polyester degradation is substantially enhanced at temperatures, above the glass transition temperature (*T*_g_) of the polymer, as this significantly increases polymer chain mobility and enzyme accessibility (Furukawa et al., 2019; Joo et al., 2018; Mueller, 2006; Roth et al., 2014; Wei & Zimmermann, 2017). Moreover, embedding enzymes into plastics requires thermal tolerance sufficient to withstand temperatures reached during molding or extrusion processes (Greene et al., 2021; Guicherd et al., 2024). Recent work has demonstrated that PET‐degrading enzymes can be engineered for increased enzyme activity and thermal tolerance (Arnal et al., 2023; Joho et al., 2024; Tournier et al., 2020; Zhang et al., 2023). Another source of thermostable enzymes is from thermophilic and hyperthermophilic microorganisms (Erickson et al., 2022; Roth et al., 2014), as their proteins are typically optimized for operation at high temperatures (Vieille & Zeikus, 2001).

Several naturally occurring bioplastic‐degrading enzymes have been identified and characterized to date (recently reviewed in Guo et al. (2024), Liu et al. (2025), Suresh et al. (2025), and Tournier et al. (2023)). This includes the PBAT degrading enzyme PpEST isolated from *P. oleovorans* (previously *Pseudomonas pseudoalcaligenes*), which showed a high thermal tolerance (Wallace et al., 2017). PBAT degradation by PpEST was observed at 80°C for up to 3 days (Wallace et al., 2017), and PpEST catalyzed hydrolysis of ionic phthalic polyesters even after 7 days of reaction (Haernvall et al., 2017).

PpEST has only a low sequence identity to other known PBAT‐degrading enzymes (Wallace et al., 2017). It has a 70% sequence identity to the structurally characterized lysophospholipase and arylesterase TesA from *Pseudomonas aeruginosa* (Wallace et al., 2017), which belongs to the SGNH‐hydrolase superfamily of serine esterases and lipases that are also referred to as GDSL‐type lipases and esterases (Kovačić et al., 2013). SGNH‐hydrolases have evolved separately from classical lipases (Akoh et al., 2004); they have their catalytic serine residue in a conserved GDSL motif at the N‐terminus whereas most classical lipases contain a centrally located GxSxG catalytic motif (Upton & Buckley, 1995). They are found ubiquitously in all organisms (Akoh et al., 2004), and are generally known to have broad substrate specificities aided by a conformationally flexible active site (Akoh et al., 2004; Lo et al., 2003; te Huang et al., 2001). Despite their relevance in polyester degradation, the structural and sequence features underpinning thermostability and activity in this enzyme family remain largely unexplored.

In this study, we have used bioinformatic analysis, including sequence similarity networks and phylogenetic approaches, to identify a distinct subfamily of bacterial SGNH‐hydrolases closely related to the PBAT‐degrading enzyme PpEST. We then selected homologs from thermophilic bacteria for experimental characterization, confirming their esterase activity and revealing their polyesterase activity toward PBAT and PBSA. During this work, we uncovered a previously unreported thermostabilizing mechanism in SGNH‐hydrolases mediated by flexible N‐terminal regions. Using biochemical assays, small angle X‐ray scattering (SAXS), X‐ray crystallography, and molecular dynamics (MD) simulations, we show that disordered N‐termini can enhance thermal resilience in this protein family, and in some homologs, also modulate oligomerization and activity.

## 2 METHODS

### 2.1 Sequence similarity networks and sequence retrieval

The PpEST sequence (Refseq ID: WP_003460012.1) was retrieved from the NCBI Reference Sequence Database (O'Leary et al., 2016). A Pfam protein families database (Paysan‐Lafosse et al., 2025) search (now integrated into InterPro; Blum et al., 2021) revealed that it was part of the GDSL‐type lipase family (Pfam ID: PF13472) within the SGNH‐hydrolase superfamily (Pfam ID: CL0264), which includes over 40,000 sequences. An initial SSN was generated using sequences from the curated UniRef50 database (Suzek et al., 2014), that retains one representative for sequences with greater than 50% similarity. The SSN was generated using the Enzyme Function Initiative Enzyme Similarity Tool (EFI‐EST) with an alignment score cut‐off of 20 (Gerlt et al., 2015), and visualized on Cytoscape 3.9.1 (Shannon et al., 2003). Nodes represented protein clusters with >40% sequence identity and edges indicated similarity based on alignment scores calculated via all‐vs‐all BLAST bit‐scores (Altschul et al., 1990; Gerlt et al., 2015). Increasing alignment score thresholds and application of a force‐directed layout (yFiles Organic Layout) allowed the identification of iso‐functional clusters once no substantial cluster changes are observed with large jumps in alignment score thresholds (Atkinson et al., 2009). The PpEST‐containing cluster was then visualized in more detail with increased alignment score cut‐offs, layout reapplication, and annotation based on organism taxonomy.

Because this first SSN used a curated set with relatively low intra‐cluster sequence identity, a second SSN was constructed with closely related PpEST homologs to maximize the representative genomes mined for thermophilic homologs. This SSN was generated and visualized similarly, but using sequences retrieved from a BLAST search against the NCBI refselect_seq database (O'Leary et al., 2016), with e‐values >0.005. Sequences from the resulting large cluster at an alignment score cut‐off >30 were combined with the PpEST cluster from the first SSN. Duplicates were removed to yield 2123 unique sequences, 1987 of which belong to the main PpEST clade. This dataset was mined for taxa with “thermo” in the name (Table S1, Supporting Information), and 10 sequences were selected based on phylogenetic proximity to PpEST and optimal growth temperature of the source organisms.

### 2.2 Phylogenetic analysis

The 10 selected sequences were collated with all the sequences from the NCBI Ref_Seq database within the PpEST cluster of the first SSN, forming a representative set of 142 sequences. A multiple sequence alignment (MSA) was generated using MUSCLE (Edgar, 2004) and MAFFT (Katoh & Standley, 2013) implemented through UGENE (Okonechnikov et al., 2012) with default settings. Any long or partial sequences were removed, and sequences re‐aligned, with iterative approximate‐maximum‐likelihood tree generation using FastTree2.1 (Price et al., 2010), until the local “SH‐like” support values for the main nodes exceeded 80%. The final MSA was used to generate a maximum‐likelihood phylogenetic tree with IQTree2 (Minh et al., 2020) using the LG + R7 evolutionary model, with UltraFast Bootstrap (UF‐Boot) support values and SH‐aLRT branch test values calculated for 1000 replicates. The tree was visualized and annotated using the Interactive Tree of Life (iTOL) webtool (https://itol.embl.de/).

### 2.3 Protein expression and purification

Signal peptides were predicted and trimmed using SignalP‐5.0 (Almagro Armenteros et al., 2019), and each trimmed sequence was cloned between the *Nde*I and *Xho*I restriction sites in the pET‐29b(+) vector for expression of C‐terminal 6xHistidine‐tagged proteins. Constructs were synthesized by GenScript (Singapore) (Table S1). The plasmids and a negative control (gfasPurple‐S125R‐F162R‐V44A‐L123T_pETcc2; Ahmed et al., 2022) were transformed into NEB T7 express cells (New England Biolabs) following the manufacturer's protocol. Fifty microliter of transformed cells were plated on Luria Broth (LB) agar plates containing 50 μg/mL of kanamycin (100 μg/mL of ampicillin for the control), incubated overnight at 37°C and stored at 4°C for up to 2 weeks.

For initial protein expression tests, single colonies were inoculated into 15 mL of auto‐induction media (Tartof & Hobbs, 1987) (5 g yeast extract, 20 g tryptone, 85.5 mM NaCl, 22 mM KH_2_PO_4_, 42 mM Na_2_HPO_4_, 0.6% glycerol, 0.05% glucose, and 0.2% lactose) containing antibiotics as above in 50 mL tubes. Cultures were shaken at 200 rpm, first for ~4 h at 37°C, and then 30°C overnight. Cultures were then either stored at 4°C (up to 2 weeks) for activity assays and protein expression was assessed using SDS‐PAGE gels (see Data S1).

For large‐scale expression, plasmids were transformed into BL21(DE3) cells (New England Biolabs). Overnight starter cultures (5 mL) were used to inoculate 1 L of the auto‐induction media and grown overnight at 200 rpm and 28°C. Cells were harvested by centrifugation (5000 × g for 10 min), resuspended in buffer (5 mM imidazole, 100 mM NaCl, 50 mM potassium phosphate pH 8.5), and lysed using a Microfluidics homogenizer M‐110P (Massachusetts, USA) at 18,000 PSI. Lysates were centrifuged (20,000 × g, 45 min, Avanti J‐E), and the soluble fraction was syringe‐filtered through a 0.22 μm filter.

The filtrate was loaded onto a 5 mL Ni‐NTA Fast Flow cartridge (Cytiva) and protein eluted with a gradient from 5 to 250 mM imidazole for 15 column volumes (CVs), followed by 5 CV at 500 mM imidazole. Elution fractions were assessed by SDS‐PAGE using NuPAGE® Novex 4%–12% Bis‐Tris gels (Invitrogen). Protein‐containing fractions were concentrated with Amicon Ultra‐15 filters and further purified using a 130 mL Superdex 200 pg. 10/300 column (Cytiva) equilibrated with 100 mM NaCl, 50 mM potassium phosphate pH 8.5. The proteins were concentrated with Amicon Ultra‐15 filters to 10–20 mg/mL before being used for crystal screens (see Data S1), snap‐frozen in liquid nitrogen and stored at −80°C. All chromatography was performed on an ÄKTA purifier UPC 10 (GE Healthcare). Analytical SEC was conducted with a Superdex 200 Increase 10/300 GL column (Cytiva), with a BioRad Gel Filtration Standard (#1511901).

### 2.4 Small angle X‐ray scattering

SAXS experiments were performed on the BioSAXS beamline at the Australian Synchrotron ANSTO (Tan et al., 2025), using the Coflow sample autoloader in SEC mode (Ryan et al., 2018), as detailed in our previous work (Ahmed et al., 2024). Briefly, 50 μL of purified protein (~10 mg/mL) was separated with a Superdex S200 5/150 GL SEC column (Cytiva), followed by UV absorbance detection and X‐ray exposure with a 12.4 keV beam (*λ* = 1.00 Å) and a Pilatus3 X 2M detector (3151 mm sample‐to‐detector distance, q‐range = 0.007–0.475 Å^−1^). Scattering data were acquired in a 1.0 mm diameter capillary, reduced using Python (PyFAI) with customized algorithms and scaled absolutely using water as a standard. Data were processed using BioXTAS‐RAW version 2.3.0 (Hopkins, 2024) with ATSAS 3.2.1 (Manalastas‐Cantos et al., 2021). BioXTAS‐RAW (Brookes et al., 2016) was used to baseline and buffer‐subtract the SEC scattering profiles and the pair distance distribution function (P(r)) was calculated using GNOM (Svergun, 1992) with AutoRG (Petoukhov et al., 2007). Molecular weights were estimated using Bayesian Inference (Hajizadeh et al., 2018), Volume of Correlation (Rambo & Tainer, 2013), and corrected Porod volume (Piiadov et al., 2019). CRYSOL (Svergun et al., 1995) was used to compare the AlphaFold3 models to the scattering profiles and DAMMIN (Svergun, 1999) was used to generate dummy atom models, which were averaged with DAMAVER and refined in DAMFILT (Volkov & Svergun, 2003). Flexible conformation ensembles were modeled using the ensemble optimized method (EOM) (Bernadó et al., 2007; Tria et al., 2015).

### 2.5 Esterase activity assay with para‐nitrophenol esters

For whole‐cell assays, 90 μL of 50 mM Tris (pH 8.0) was first mixed with 5 μL of cell culture in a 96‐well plate (Grenier #655101). The substrate pNP‐butyrate (MW ~ 209 Da) is sufficiently small and amphiphilic to diffuse through *Escherichia coli* outer‐membrane porins (OmpF/OmpC) and the lipid bilayer, allowing intracellular enzyme activity to be detected without intentional lysis (Delcour, 2009; Deylami et al., 2024). For purified enzymes, 5 μL of protein was added instead to 90 μL of 50 mM Tris (pH 8.5) to reach final concentrations of 50–600 nM (410, 200, 95, 51, 129, 72, 591 nM for Ct_EST, Ft_EST, Mt_EST, Pmt_EST, Rp_EST, St_EST, and Tt_EST, respectively). Reactions were initiated by adding 5 μL of 15 mM stock (in 100% methanol) of pNP‐acetate, ‐propionate, ‐butyrate, ‐valerate, ‐octanoate, or ‐decanoate, or 15 μL of 5 mM pNP‐myristate, resulting in 5% methanol (15% for pNP‐myristate due to its low solubility in a 5% methanol aqueous solution). Absorbance at 410 nm was measured every 3–4 s over 10 min, and the slope from the linear region (first 30–100 s) was used to calculate pNP release via a standard curve.

For kinetic assays, pNP‐butyrate stocks in 100% methanol were used to achieve final concentrations of 1000, 750, 500, 250, 150, 100, and 50 μM in reactions with 5% methanol and 0.05–0.1 μM enzyme. Reaction rates (μM pNP/s) were divided by enzyme concentration to obtain *k* (s^−1^). Reactions were performed in triplicate, with technical duplicates. Michaelis–Menten curves were generated in GraphPad Prism v10 to calculate *K*_M_ and *k*_cat_ with symmetrical confidence intervals for standard error estimation.

### 2.6 Thermostability assay

For initial thermostability assessment, 8 × 30 μL aliquots of cell culture per enzyme were heated to various temperatures for 10 min in 0.2 mL tubes (strips) using the gradient function on a thermocycler (Kyratec Supercycler). Tubes were cooled on ice for 10 min, and residual activity was measured in 96‐well plates by adding 5 μL of each suspension to 90 μL of 50 mM Tris (pH 8.0), followed by 5 μL of 15 mM pNP‐butyrate in 100% methanol to initiate the reaction. For each enzyme, 5 μL of unheated culture and 5 μL of buffer were used as positive and negative controls, respectively. Reaction rates (OD/min) were used to calculate residual activity as a percentage of the unheated control. This process was also used for the purified proteins, where 55–100 μL aliquots (0.2–0.5 μM) were heated for 10 min, cooled on ice, then diluted to 95 μL with buffer (50 mM Tris pH 8.0) in a 96‐well plate for a final concentration of 0.2 μM. Reactions were initiated with 5 μL of substrate.

To measure time‐dependent stability at 99°C, 0.5 μM and 25 μM solutions of purified enzymes were prepared in 50 mM Tris (pH 8.0), and 8 × 20 μL aliquots were placed in 0.2 mL tubes. Tubes were incubated at 99°C, and one was removed at each time point (0, 5, 10, 15, 30 min; 1, 2, and 4 h), then cooled on ice. Samples were diluted to 0.1 μM and assayed for residual activity with pNP‐butyrate as described above, using the 0 min sample as the positive control. *T*₅₀ (time to 50% activity loss) was calculated in GraphPad Prism by fitting a two‐phase decay curve. All experiments were performed in triplicate.

### 2.7 PBAT and PET degradation assays

PBAT (SolPol 1000 N, Cosmos Plastics and Chemicals) and PBSA (BioPBS FD92, PTTMCC) samples were ground under liquid nitrogen using an IKA Lab Mill, vacuum dried at 50°C and sieved to 200–400 μm. Amorphous PET samples (≤500 μm) were prepared as previously described (Joho et al., 2024).

Polyester degradation assays were performed in 500 μL reactions (2 mL tubes) with 5 mg/mL suspensions of PBAT, PBSA, or PET in 50 mM Tris (pH 8.0). Reactions were initiated by adding 10 μL of cell suspension (for whole‐cell assays) or 0.5–1 μM purified enzyme. For PBAT degradation by PpEST, 0.25, 0.5, 1, and 2 μM were tested. A negative control (buffer and plastic only) was also included. Reactions were incubated at room temperature, 40°C, or 70°C, shaking at 800 rpm for 24, 48, or 96 h.

After incubation, samples were centrifuged at 16,000 × g for 1 min to pellet residual plastic. Two 200 μL aliquots of each supernatant were transferred to a 96‐well UV plate (Grenier #655801), and absorbance at 250 nm was measured using a Spectramax Plus 384 spectrophotometer (Molecular Devices). Terephthalic acid equivalents were quantified using a standard curve. Aliquots were returned to their tubes for continued incubation, minimizing evaporation. Technical replicates were averaged, and 2–3 biological replicates were used to calculate standard error.

To detect 1,4‐butanediol from PBAT or PBSA degradation, 10 μL of 5 mM NAD^+^ and 5 μL of 4 U/mL equine alcohol dehydrogenase (Sigma‐Aldrich #55689‐100MG) were added to 185 μL of each sample. NADH production was monitored at 340 nm over 10 min and quantified against a 1,4‐butanediol standard curve.

### 2.8 CD spectroscopy

One hundred microliter esterases were dialyzed with 10 kDa MWCO Slida‐A‐Lyzer mini devices (ThermoFisher) against 1 L of 10 mM potassium phosphate pH 7.2, at 4°C, overnight. The samples were diluted to approximately 6 μM with the same potassium phosphate buffer and loaded into 1 mm quartz QS cuvettes (Hellma). Spectra were collected in a J‐1500 circular dichroism spectrometer (Jasco) with a Peltier‐controlled cell holder set to 25°C from 180 to 260 nm with a scanning speed of 20 nm/min, 1.00 nm bandwidth, 1 s digital integration time, and five accumulations per spectrum. Measurements over 700 V for the high‐tension voltage channel were excluded from analysis. Melt experiments were carried out by measuring spectra at each degree from 25°C to 95°C with a temperature ramp of 1°C/min, holding at each temperature for 1 min before measurement. Spectra collected during melt experiments were taken with a scanning speed of 100 nm/min, 1.00 nm bandwidth, 0.5 s digital integration time, with only one accumulation per spectrum. Melting temperatures were determined with a Boltzmann sigmoidal fit to ellipticity values at a specific wavelength as a function of temperature in GraphPad Prism. Errors of the fitting to the inflection point as the melting temperature are displayed with their 95% confidence interval.

### 2.9 Molecular dynamics simulations

Alphafold3 structures of Ct_EST, shCT_EST, Mt_EST, and shMT_EST were used as starting models for the MD simulations. Each system was simulated in triplicate for 1000 ns using AMBER22 (Case et al., 2020), with the FF19SB forcefield and the TIP3P solvation model (Tian et al., 2020). Structures were solvated in a periodic octahedral water box with a 12 Å buffer and charge neutralized with Na^+^ ions. Energy minimization was performed in 25,000 steps of steepest descent with positional backbone restraints. Equilibration was then conducted using the NTP ensemble with restrained backbone atoms, a Berendsen barostat (1 atm, 2 ps relaxation), and a Langevin thermostat (298 K). SHAKE constraints were applied to hydrogen‐containing bonds with a 12 Å cutoff for non‐bonded interactions. Trajectories were saved every 1000 steps (2 ps) for 1 ns, producing 200 frames. Production runs (1000 ns) used identical parameters but without backbone restraints, saving 100,000 frames (every 5000 steps). Trajectories were analyzed using CPPTRAJ (AMBER22) and in‐house Python scripts. For clustering, backbone‐aligned frames from equilibrated segments (typically 200–1000 ns) were clustered by RMSD of N‐terminal residues 1–25 (Ct_EST) or 1–12 (Mt_EST), using hierarchical agglomerative clustering on every 10th frame. RMSD cutoffs ranging from 2 to 6 Å were tested and both representative and averaged cluster structures were generated.

## 3 RESULTS

### 3.1 PpEST belongs to a distinct family of bacterial SGNH‐hydrolases

Initially, we used a sequence similarity network (SSN) to distinguish closely related proteins to PpEST that are likely to have polyesterase activity from other members of the SGNH‐hydrolase superfamily. In this SSN, each node represents a cluster of proteins with more than 40% sequence similarity, and lines between the nodes represent the similarity quantified as an alignment‐score (Gerlt et al., 2015). The progressive increase in the alignment score cut‐off, followed by application of a force‐directed layout, allows the identification of potentially iso‐functional protein clusters (Ahmed et al., 2015; Atkinson et al., 2009; Gerlt et al., 2015; Uberto & Moomaw, 2013).

**FIGURE 1:**
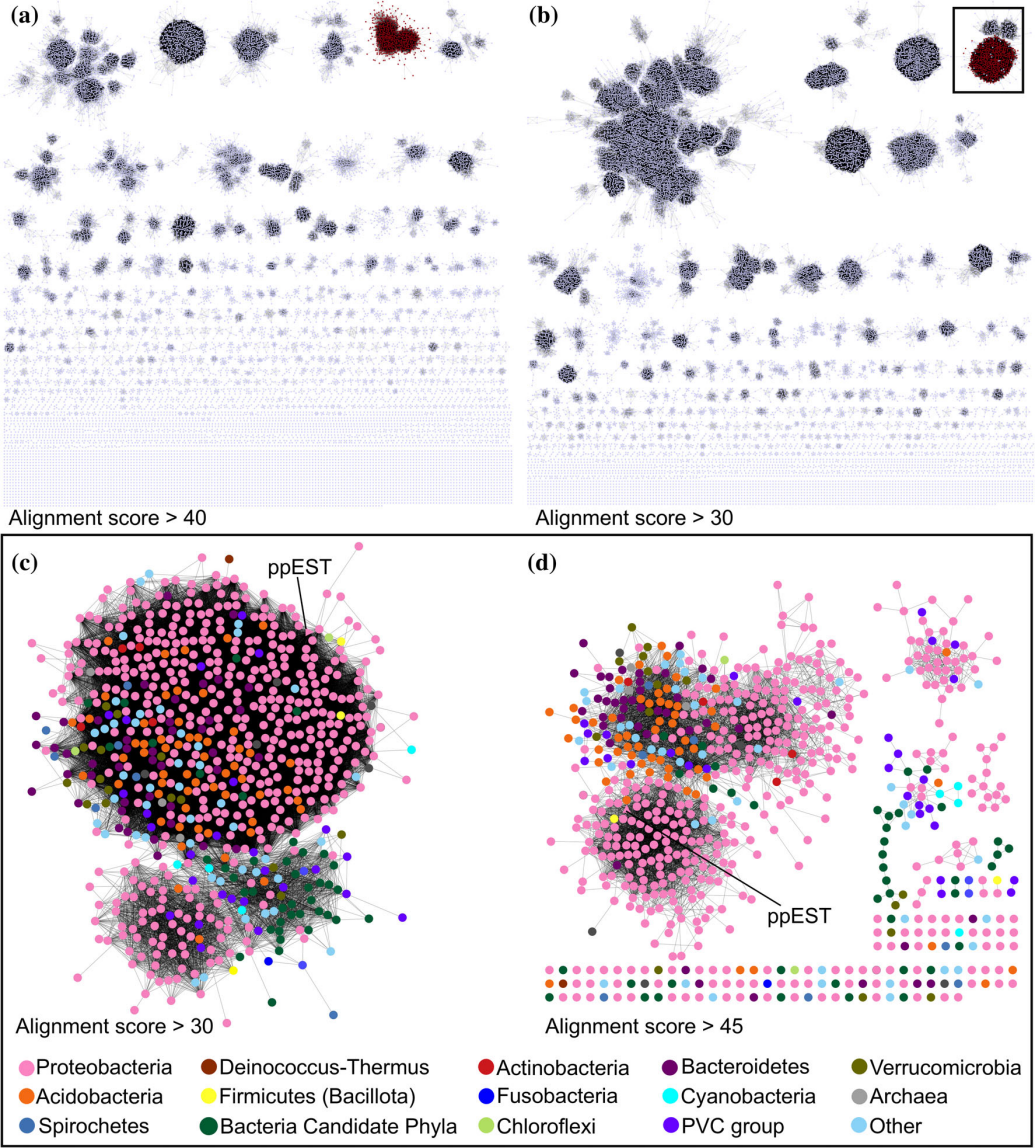
Sequence similarity network (SSN) of the GDSL‐type lipase family within the SGNH‐hydrolase superfamily. Each node represents a cluster of proteins with >40% sequence similarity. Edges represent the similarity between nodes, quantified by the alignment score calculated using the EFI‐EST webtool (Gerlt et al., 2015). (a and b) The SSN of the GDSL‐type lipase family at alignment score cut‐offs of 40 and 30, respectively. The PpEST subfamily cluster is highlighted in red. (c and d) A detailed view of the clusters marked in a box in (b), at alignment score cut‐offs of 30 and 45, respectively. Nodes are colored according to phyla, as indicated in the provided key. The node for PpEST is labeled and represented as a triangle.

At an alignment‐score cut‐off of 40 (approximately equal to a BLAST log*E*‐value of −40; Altschul et al., 1990; Gerlt et al., 2015; Figure 1a), the PpEST‐like sequences formed a distinct cluster. This cluster is closely related to two smaller clusters, as seen at a less stringent alignment score cut‐off of 30 (Figure 1b), which were also conservatively included in further bioinformatic analysis. Together, these clusters remained distinct from other nodes, even at lower alignment‐score cut‐off values of 25 and 27 (Figure S1), suggesting a functionally distinct group or subfamily of proteins.

This PpEST cluster predominantly contained sequences from the phylum Proteobacteria (Pseudomonadota), with representation also from Acidobacteriia and Bacteroidetes (Figure 1c). Sequences from the bacterial candidate phylum clade were also present, which represent organisms from metagenomic sequencing of uncultured samples (Brown et al., 2015). At an alignment score cut‐off of 30, PpEST lies within a central cluster that resolves into three subclusters at a cut‐off of 45 (Figure 1d), where PpEST's sub‐cluster almost exclusively contains sequences from Proteobacteria.

**FIGURE 2:**
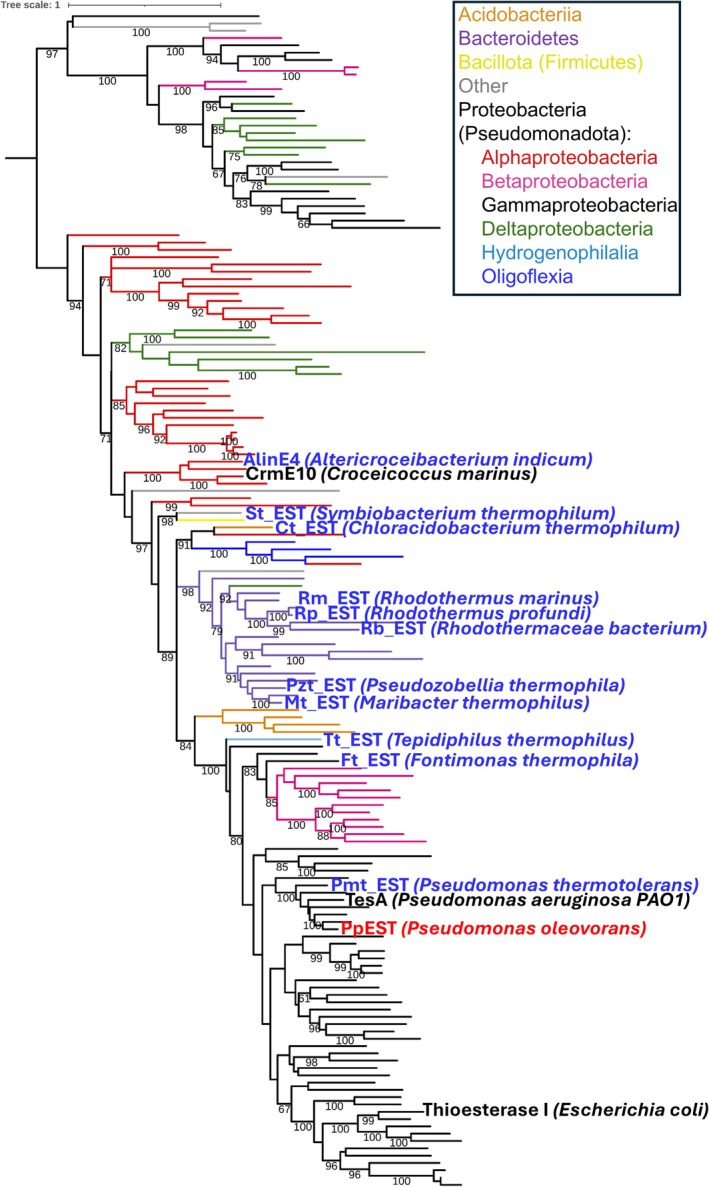
Phylogenetic tree of the PpEST sub‐family (boxed in Figure 1b), with PpEST highlighted in red. The proteins selected for characterization in this study are presented in blue, and any other characterized protein sequences in this set are highlighted in black. Tree branches are colored as per phylogeny as indicated in the provided key. Ultra‐fast bootstrap support values (UF‐BOOT) are shown for nodes with UF‐BOOT >60% and SH‐aLRT branch test values >80%.

Like the SSN, a phylogenetic tree of representative sequences from the main PpEST cluster and the two related small clusters (excluding sequences from metagenomes) (Figure 1c), revealed a large main clade and a separate smaller clade (Figure 2). Most of the represented organisms in the separate smaller clade are also present in the main clade (Figure 2), suggesting that while these sequences are closely related to PpEST, they may have resulted from gene duplication and can potentially be functionally divergent. Hence only sequences from the larger clade were considered as the PpEST protein family for further analysis.

The phylogenetic tree showed that PpEST is most closely related to a subclade of proteins from Gamma Proteobacteria (Figure 2). This clade primarily comprises sequences from aquatic bacteria, including species of *Pseudomonas*, *Marinobacter*, *Shewanella*, *Pseudoalteromonas*, and *Legionella*. Other Proteobacteria (Pseudomonadota) classes, including Alpha Proteobacteria, Beta Proteobacteria, Delta Proteobacteria, Oligoflexia, and Hydrogenophilalia were also represented in closely related subclades, as well as sequences from the phyla Bacteriodetes and Acidobacteriia. Sequences from other phyla, such as Firmicutes (Bacillota), were also present at a lower frequency, most likely acquired through horizontal gene transfer.

In addition to TesA from *P. aeruginosa* (Wallace et al., 2017), which is closely related to PpEST, we found four other structurally characterized esterases within the PpEST family. These are the medium‐chain esterase EstA from the cold‐adapted bacterium *Pseudoalteromonas* sp. *643A* (PDB ID: 3HP4) (Brzuszkiewicz et al., 2009; Cieśliński et al., 2007), Thioesterase I from *E. coli* that exhibited activities with thioesters, esters, arylesters, proteins, and lysophospholipids with a stereoselectivity for amino acid‐derived substrates (PDB ID: 1JRL) (Lo et al., 2003), and the esterases AlinE4 (PDB ID: 7C2A) and CrmE10 (PDB ID: 7C23) from *Altererythrobacter indicus* and *Croceicoccus marinus*, respectively (Li et al., 2020). Of these characterized enzymes, AlinE4 possessed remarkable thermostability with residual activities detected even after 2 h of incubation at 100°C (Li et al., 2020).

### 3.2 PpEST homologs can hydrolyze pNP‐esters with a preference for pNP‐butyrate

We then selected potentially thermostable homologs of PpEST from thermophilic bacteria for experimental characterization. Ten candidates were selected based on the growth temperature tolerance of the source organism (maximum growth temperature >50°C) and protein sequence similarity to PpEST. The sequences were Pmt_EST from *Pseudomonas thermotolerans* (WP_027897298.1), Ft_EST from *Fontimonas thermophila* (WP_091533759.1), Tt_EST from *Tepidiphilus thermophilus* (WP_082438342.1), Mt_EST from *Maribacter thermophilus* (WP_047245951.1), Pzt_EST from *Pseudozobellia thermophila* (WP_072993541.1), Rb_EST from *Rhodothermaceae bacterium* (MYD18970.1), Rp_EST from *Rhodothermus profundi* (WP_072716363.1), Rm_EST from *Rhodothermus marinus* (WP_012843053.1), Ct_EST from *Chloracidobacterium thermophilum* (WP_014100090.1), and St_EST from *Symbiobacterium thermophilum* (WP_011195153.1). Due to its previously reported high thermal stability, we also included the esterase AlinE4 (PDB ID: 7C2A) (Li et al., 2020), although the source organism *A. indicus* is mesophilic (Kumar et al., 2008). For each selected sequence, SignalP5.0 (Almagro Armenteros et al., 2019) was used to computationally predict and remove any signal sequences at the N‐terminus, because native secretion signals are specific to their original hosts and are generally not processed efficiently by the *E. coli* secretion systems. Their retention can reduce soluble expression or lead to the accumulation of misfolded or unprocessed protein (Choi & Lee, 2004; Freudl, 2018; Jomrit et al., 2023). A C‐terminal 6x‐Histidine tag was added to the codon optimized sequence prior to the generation of protein expression vectors for *E. coli* strain BL21DE3 (Table S1).

**FIGURE 3:**
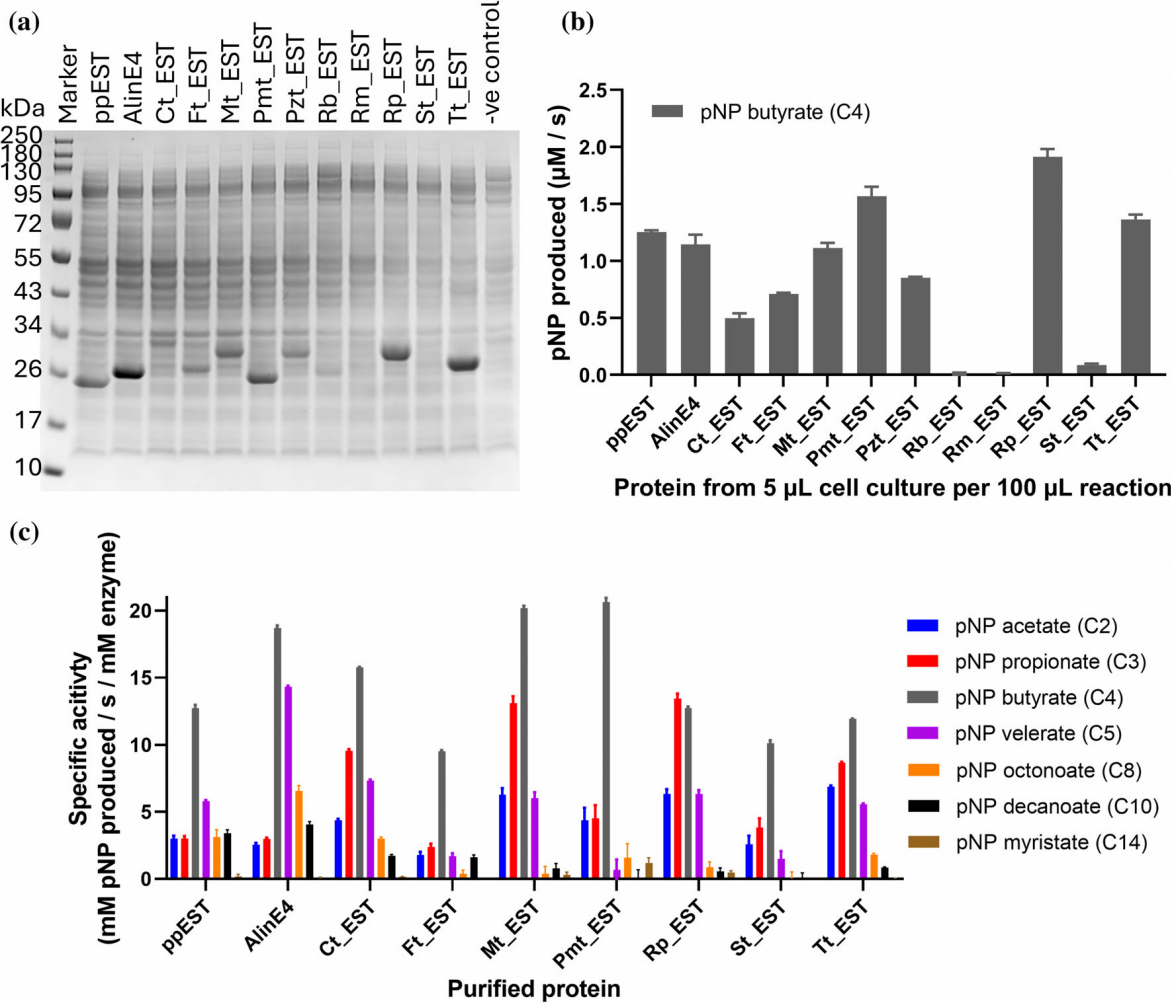
Expression and esterase activity of PpEST homologs. (a) SDS‐PAGE gel of soluble proteins expressed from small‐scale expression tests of PpEST homologs in *E. coli* BL21(DE3) cells. Gels of purified proteins are shown in Figure S3. Expected molecular weights are: PpEST (20.2 kDa), AlinE4 (20.6 kDa), Ct_EST (25.1 kDa), Ft_EST (21.3 kDa), Mt_EST (24.7 kDa), Pmt_EST (20.5 kDa), Pzt_EST (23.6 kDa), Rb_EST (20.5 kDa), Rm_EST (24.1 kDa), Rp_EST (24.6 kDa), St_EST, Tt_EST (21.9 kDa). (b) Esterase activity by cell culture expressing PpEST homologs with pNP substrates of increasing lengths. Assays were performed in triplicate 100 μL reactions at pH 8.0, and error bars represent the standard error from the mean (SEM). (c) Esterase activity by purified PpEST homologs with pNP substrates of increasing length. Assays were performed in 100 μL reactions at pH 8.5, and error bars represent the standard deviation from the mean (SD).

Small‐scale protein expression tests revealed bands of soluble proteins of the correct apparent molecular weights on SDS‐PAGE gels for all proteins except Rm_EST and St_EST (Figure 3a). To confirm protein expression, we also tested whole cell samples from each cell culture for esterase activity against p‐nitrophenyl (pNP) butyrate (C4), which had been previously shown to be the carboxyl ester substrate with the highest rates of hydrolysis by PpEST (Wallace et al., 2017) and AlinE4 (Li et al., 2020; Wallace et al., 2017). While most of the proteins showed activity with pNP butyrate, no activity was detected for the cell extracts of *E. coli* expressing Rm_EST, and only low‐level activity was detected for St_EST. Rb_EST also had no detectable activity with pNP butyrate, despite the positive indication of soluble protein expression in the SDS‐PAGE gels.

Because whole‐cell assays provided only a crude measure of activity, with variable protein levels across samples, we also performed large‐scale protein expression followed by nickel affinity chromatography. All proteins were successfully purified, except for Rb_EST, Rm_EST, and Pzt_EST, which were not characterized further. Comparison of the specific activities of the purified proteins with different lengths of pNP substrates that were previously tested for PpEST and AlinE4 (C2, C3, C4, C5, C8, C10, and C14), showed a clear preference for mid‐chain length esters, particularly pNP‐butyrate (C4) (Figure 3c), as had been observed previously for PpEST (Wallace et al., 2017) and AlinE4 (Li et al., 2020; Wallace et al., 2017). The overall activities of all enzymes were comparable to PpEST and AlinE4.

### 3.3 An extended N‐terminus increases thermostability in Ct_EST, Mt_EST, and Rp_EST

Next, we narrowed down our selected sequences to the most thermostable candidates. An initial thermostability assessment was performed, measuring the residual activity with pNP‐butyrate following a 10 min heat treatment using both whole‐cell and purified enzyme samples (Table 1, Figure S2). AlinE4 and Rp_EST retained residual activities of 70% and 25%, respectively, in whole‐cell assays at 99°C, and 40% and 65% when tested as purified proteins. Ct_EST and Mt_EST retained 20% and 15% activity in whole‐cell assays and 60% and 15%, respectively, in the purified protein assays. Although PpEST and St_EST both showed 30% residual activity at 99°C when purified but not in whole‐cell assays, St_EST was excluded from further analysis due to poor purification yields. While results varied somewhat between the assay formats, AlinE4, Ct_EST, Mt_EST, and Rp_EST demonstrated the highest thermal tolerance and were selected for further characterization, along with PpEST, which was also included for comparison.

**TABLE 1:** Summary of residual activity of the enzymes after heating at 99°C for 10 min, summarizing data provided in Figure S2.

Protein	Residual activity after 10 min at 99°C (%)	
	Cell culture (*n* = 2)	Purified protein (0.2–0.5 μM, *n* = 3)
PpEST	0	26.0 ± 2.6
AlinE4	69.8 ± 5.2	37.1 ± 0.6
Ct_EST	19.7 ± 0.8	61.3 ± 4.9
*shCt_EST*	*n/a*	*4.6* ± 0.4
Ft_EST	0	1.0 ± 4.1
Mt_EST	16.6 ± 0.7	13.8 ± 2.0
*shMt_EST*	*n/a*	*15.1* ± 1.2
Pmt_EST	19.1 ± 0.1	5.1 ± 2.7
Rp_EST	27.9 ± 2.1	63.8 ± 1.1
*shRp_EST*	*n/a*	*10.5* ± 1.2
St_EST	0	24.4 ± 3.6
Tt_EST	0	2.3 ± 0.8
Pzt_EST	0	n/a

*Note*: Enzymes that were not measured at 99°C because of complete activity loss at lower temperatures are marked as having 0% activity. N‐terminal truncated proteins are highlighted in gray and the standard error is provided.

Sequence comparison revealed that Ct_EST, Mt_EST, and Rp_EST each contain extended N‐terminal regions that are not present in AlinE4 or PpEST. These N‐terminal regions were not predicted to be part of export signals and were poorly conserved between the three proteins (Figure S4). They contained a high proportion of serine, threonine, proline, lysine, and glutamate residues with almost no bulky aromatic residues, which is commonly the case in intrinsically disordered regions (IDRs) (Van Der Lee et al., 2014). To determine whether these regions contribute to thermal stability, we also expressed and purified shorter versions of these proteins, with their N‐termini truncated to align with AlinE4 and PpEST (shCt_EST, shMt_EST, and shRp_EST). Both shCt_EST and shRp_EST displayed reduced thermostability, retaining only 5% and 10% residual activity, respectively, after heating at 99°C for 10 min, compared to 60% and 65% for their full‐length counterparts (Table 1 and Figure S2). In contrast, shMt_EST retained similar thermal tolerance to full‐length Mt_EST. Notably, SDS‐PAGE revealed that both Mt_EST andshMt_EST may undergo autohydrolysis due to the presence of a band of the expected size as well as a second smaller band in the purified protein samples (Figure S3), which may suggest partial removal of the extra N‐terminal region.

**FIGURE 4:**
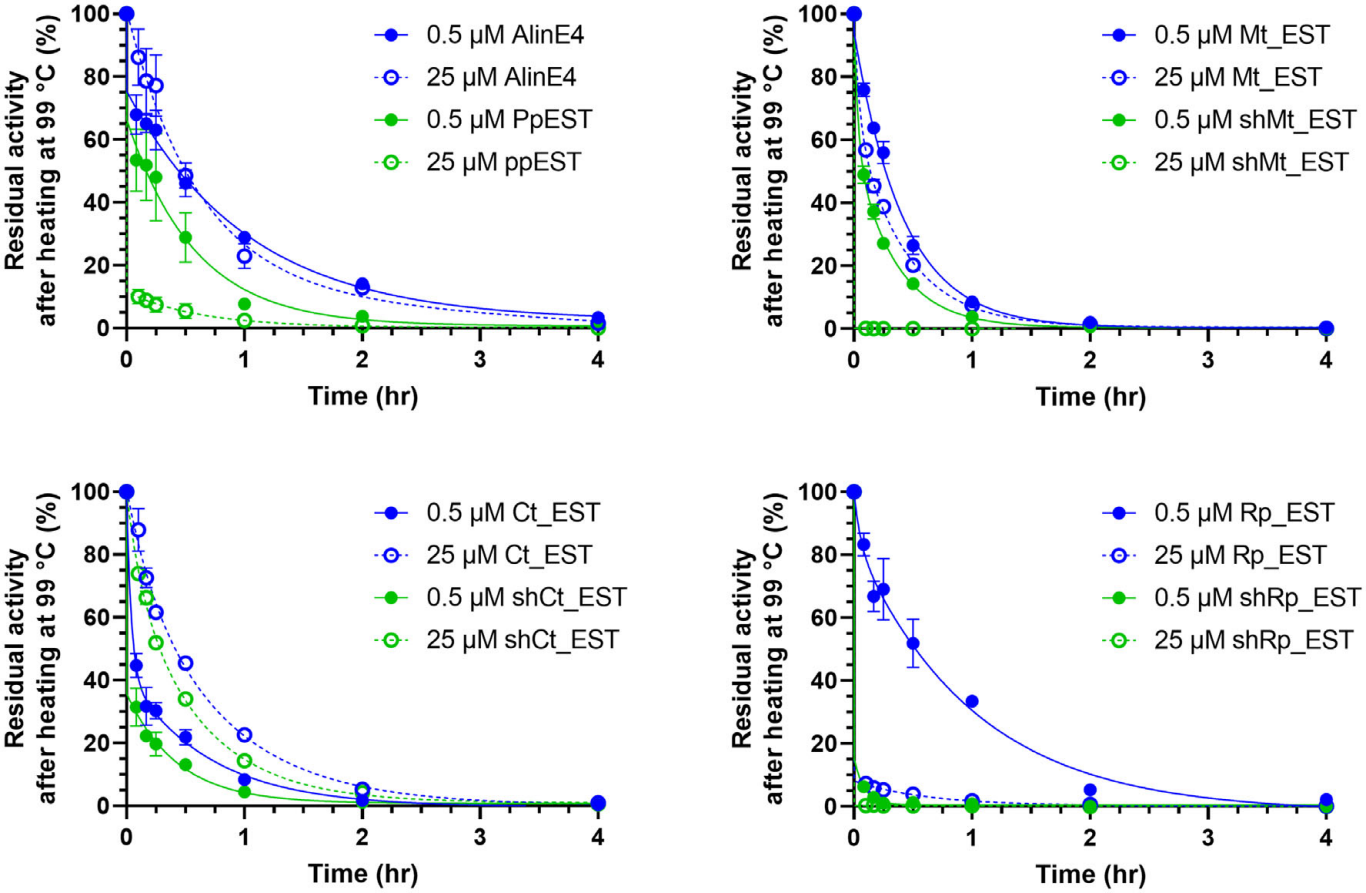
Thermostability of PpEST, AlinE4, Ct_EST, shCT_EST, Mt_EST, shMt_EST, Rp_EST, and shRp_EST when heated at 99°C, at low (0.5 μM) and high (25 μM) protein concentrations. The data (*n* = 3) are fitted to a two‐phase decay curve.

We next assessed the thermostability of these proteins over time at 99°C using two different protein concentrations: 0.5 and 25 μM (Table 2 and Figure 4). All proteins except Rp_EST and shRp_EST retained some residual activity after 1 h of heating at both concentrations. For Rp_EST, residual activity was only observed at 0.5 μM enzyme, suggesting concentration‐dependent aggregation or instability (Figure 4).

**TABLE 2:** The thermostability of the most robust PpEST homologs identified in this study and their N‐terminus truncated versions.

Enzyme	*T*_50_ at 99°C (h:min)		*T*_m_ CD 222 nm (°C)
	0.5 μM	25 μM	
PpEST	0:01	<0:05	59.59 ± 0.74
AlinE4	0:27	0:30	60.94 ± 0.58
Ct_EST	0:04	0:25	77.50 ± 2.39^a^
shCt_EST	<0:05	0:17	74.45 ± 2.15^a^
Mt_EST	0:16	0:08	57.03 ± 0.43
shMt_EST	0:05	<0:05	51.99 ± 0.56
Rp_EST	0:31	<0:05	>95^a^
shRp_EST	<0:05	<0:05	>95^a^

*Note*: The *T*_50_ (time to reduce activity by 50%) after heating at 99°C (Figure 4) is summarized as well as *T*_m_ (melting temperature) from CD spectroscopy, where errors indicate the 95% confidence intervals of the nonlinear fit used to determine *T*_m_ values.

^a^ Does not completely unfold at 95°C, so the *T*_m_ could not be accurately determined.

Among all proteins, AlinE4 exhibited the greatest thermal resilience, with a T_50_ (time to 50% loss of activity) of 27 and 30 min at 0.5 and 25 μM, respectively (Table 2 and Figure 4). Rp_EST also showed high stability at 0.5 μM (*T*_50_ = 31 min), but this was not maintained at 25 μM, where activity dropped sharply within 5 min. A similar trend was observed for PpEST, Mt_EST, and shMt_EST, all of which displayed greater thermal stability at the lower concentration. By contrast, Ct_EST and shCt_EST performed better at the higher protein concentration, possibly indicating a different unfolding mechanism compared to the other enzymes. AlinE4 retained ~20% activity even after 2 h at 99°C, with no concentration dependence (Figure 4).

Compared to the full‐length protein, the truncated shRp_EST showed no detectable activity after just 5 min at 99°C at either concentration (Figure 4). ShCt_EST and shMt_EST also showed reduced stability compared to their full‐length forms, though the effect was less pronounced (Table 2 and Figure 4). These data suggest that the additional N‐terminal residues enhance protein stability at high temperatures.

To determine whether the observed differences in thermostability correlated with changes in folding or structural stability, we performed circular dichroism (CD) spectroscopy with these purified enzymes (Table 2). CD spectra at 20°C confirmed that all proteins were folded, displaying characteristic minima at 208 and 222 nm indicative of α‐helical content (Figure S5). Thermal denaturation curves revealed similar melting temperatures (*T*_m_) for PpEST and AlinE4 (~60°C), while Mt_EST showed a slightly lower *T*_m_ (57°C) that dropped further to 52°C in shMt_EST, consistent with a destabilizing effect from truncation. Notably, both Rp_EST and shRp_EST showed very little change in ellipticity with temperature and no clear melting transition (Figure S5), suggesting that they remain folded even at 95°C, rendering the *T*_m_ undeterminable. Ct_EST and shCt_EST also displayed high thermal stability with incomplete unfolding at 95°C (Figure S5), yielding estimated *T*_m_ values of 77.5°C and 74.5°C, respectively. Although CD spectroscopy indicated that shRp_EST remained largely folded up to 95°C after a 1 min heating step, it showed markedly lower residual activity after 10 min at 99°C. This difference likely reflects the longer incubation time and differing buffer conditions used in the activity assays (50 mM Tris, pH 8.0 vs. 10 mM phosphate, pH 7.2), as buffer composition and ionic strength can influence protein stability and aggregation under heat stress.

### 3.4 The extended N‐terminal region does not always cause protein oligomerization or affect enzyme activity

**FIGURE 5:**
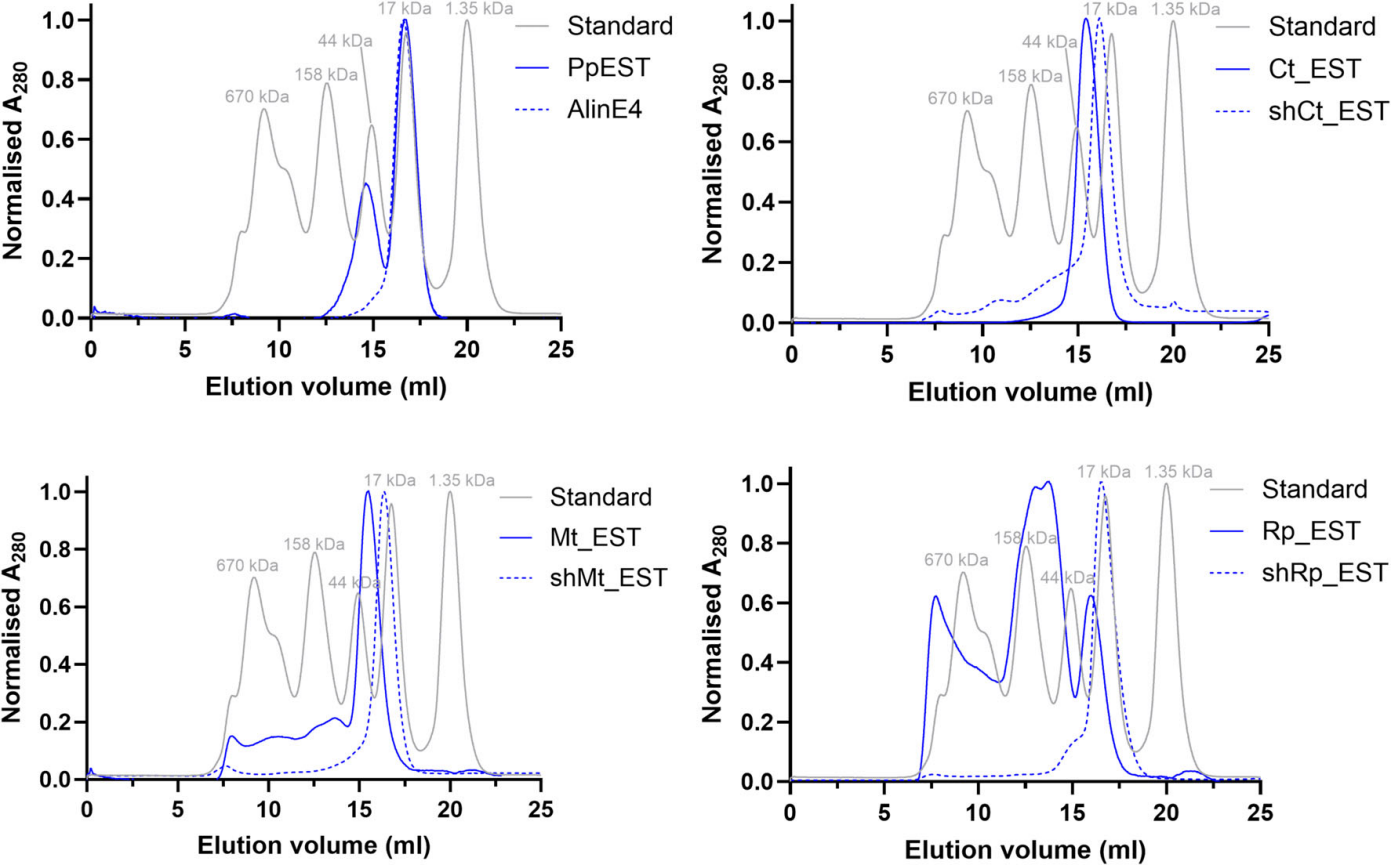
Normalized elution profiles of PpEST, AlinE4, Ct_EST, shCT_EST, Mt_EST, shMt_EST, Rp_EST, and shRp_EST from analytical size exclusion chromatography. A standard (BioRad Gel Filtration standard) is also shown for comparison and molecular weight estimates are provided in Table S3.

To understand how an extended N‐terminal region could enhance thermal resilience, we investigated if there is any effect on protein oligomerization, which is well known to affect protein thermal stability, often improving their thermal tolerance (Giuliani et al., 2007; Vieille & Zeikus, 2001). Hence, we performed analytical size exclusion chromatography (SEC) to assess differences in oligomerization between the full‐length and truncated constructs. We found that shMt_EST, Mt_EST, shCt_EST, Ct_EST, shRp_EST, and AlinE4 are most likely monomers in solution under the conditions tested (Figure 5, Table S3). This is despite previous observations that AlinE4 formed a domain‐swapped dimer in its crystal structure (Li et al., 2020), suggesting that the oligomeric state in solution may differ from that in the crystal structure. Rp_EST showed a mixture of monomeric, dimeric, tetrameric, and aggregated protein, and PpEST showed peaks for both monomeric and dimeric protein despite not containing an extended N‐terminus. Hence, while the extra N‐terminal sequences cause oligomerization in Rp_EST, this is not the case for Ct_EST or Mt_EST tested in this work.

We then tested whether the N‐terminal regions affect catalytic function (Table 3). Activity assays with pNP‐butyrate at room temperature revealed that both full‐length and truncated variants of Ct_EST and Mt_EST retained similar catalytic activity (*k*_cat_) and substrate affinity (*K*_M_). This suggests that the N‐terminus does not affect the enzymatic activity when pNP‐butyrate is used as a substrate. Interestingly, Rp_EST exhibited a ~2‐fold increase in catalytic activity (*k*_cat_) when truncated, with no change in substrate affinity (*K*_M_) (Table 3). Since the presence of the N‐terminus also causes oligomerization of Rp_EST (Table S3), oligomerization may be the reason for the lower activity of the full‐length protein compared with the truncated version.

**TABLE 3:** *In vitro* pNP‐butyrate hydrolysis activity of AlinE4, PpEST, Ct_EST, shCt_EST, Mt_EST, shMt_EST, Rp_EST, and shRp_EST.

	*k*_cat_ (s^−1^)	*K*_M_ (μM)	*k*_cat_/*K*_M_ (s^−1^ M^−1^)
AlinE4	15.0 ± 1.4	194 ± 55	7.7 × 10^4^
PpEST	14.0 ± 1.0	242 ± 49	5.8 × 10^4^
Ct_EST	38.5 ± 4.8	791 ± 178	4.9 × 10^4^
shCt_EST	33.3 ± 3.7	646 ± 139	5.2 × 10^4^
Mt_EST	54.4 ± 4.4	453 ± 81	1.2 × 10^5^
shMt_EST	53.2 ± 4.4	419 ± 78	1.3 × 10^5^
Rp_EST	12.0 ± 1.3	246 ± 72	4.9 × 10^4^
shRp_EST	25.0 ± 1.6	257 ± 43	9.7 × 10^4^

*Note*: Assays (*n* = 3) were performed at room temperature in 50 mM Tris pH 8.0, with 0.05–0.1 μM of the respective enzyme. The errors for *k*_cat_ and *K*_M_ presented were calculated during curve fitting using GraphPad Prism, and the plots are shown in Figure S14.

### 3.5 The N‐terminal region is conformationally flexible and likely disordered

To further understand the increased thermal resistance due to the extended N‐termini in these proteins, we next investigated the structures of these proteins. We initially modeled the protein structures of the full‐length (Ct_EST, Mt_EST, and Rp_EST) and truncated (shCt_EST, shMt_EST, shRp_EST, PpEST, and AlinE4) proteins using AlphaFold3 available through the AlphaFold Server (Abramson et al., 2024). For the three full‐length proteins, the N‐terminal region was predicted to be unstructured in monomeric or dimeric models and exhibited a very low confidence as measured by pLDDT (Figure S6). While this may be a result of a lack of empirical structures of SGNH‐hydrolases with similarly extended N‐termini in the databases used for model training, these features are also typical for intrinsically disordered regions (IDRs) (Ruff & Pappu, 2021). Consistently, IDR prediction using Metapredict (Lotthammer et al., 2024) and PrDOS (Ishida & Kinoshita, 2007) also supports the classification of the N‐terminus of the full‐length proteins as likely IDRs (Figure S7). Only AlinE4 was predicted to be dimeric in a domain‐swapped configuration, as observed in its crystal structure (Li et al., 2020).

Consistent with unstructured N‐terminal regions, the CD spectra of the full‐length and truncated Ct_EST, Mt_EST, and Rp_EST demonstrated that the full‐length constructs displayed slightly more negative ellipticity below 200 nm than their truncated counterparts (Figure S5). This may suggest the presence of more disordered regions, which typically exhibit negative ellipticity near 195 cm (Micsonai et al., 2015; Venyaminov et al., 1993). The lack of structure for the extended N‐termini is also supported by secondary structure estimates from BeStSel (Micsonai et al., 2015), predicting slightly higher proportions of unfolded (other) content in the full‐length proteins (Table S2).

**FIGURE 6:**
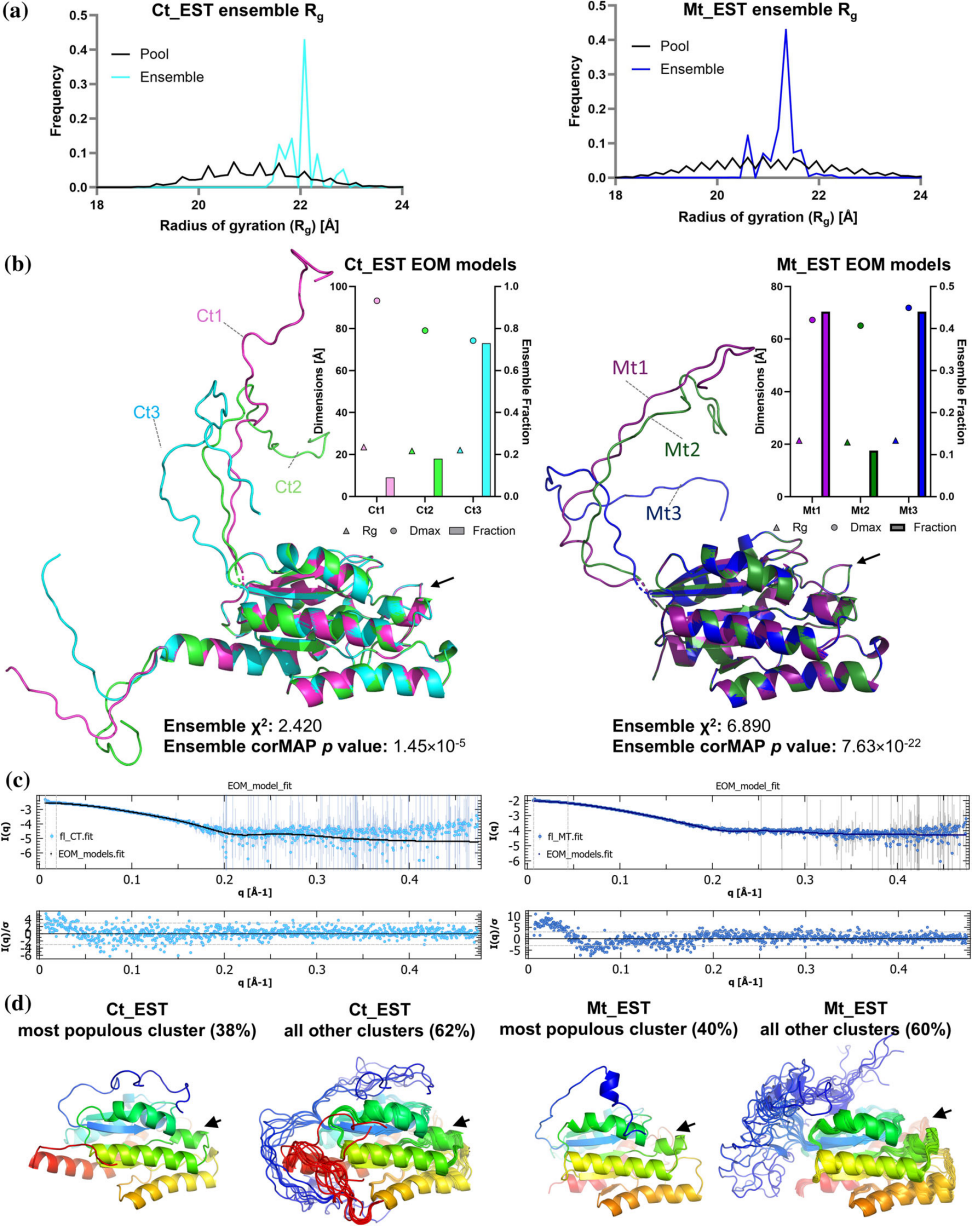
Conformational ensembles of Ct_EST and Mt_EST from SEC‐SAXS analysis and MD simulations. (a) Radius of gyration (Rg) distributions from ensemble optimized modeling (EOM) for Ct_EST (left) and Mt_EST (right), showing the initial pool (black) and selected ensemble (colored) conformers. (b) Structural overlayed ensemble of three representative EOM models for Ct_EST (left) and Mt_EST (right), with black arrows indicating the active site position. The inset shows a summary of the model dimensions (*R*_g_ and *D*_max_) and their fractional contributions to the ensemble. (c) Experimental SAXS profiles (points) and EOM fits (black lines) for Ct_EST (left) and Mt_EST (right), with residuals shown below. (d) Averaged structures from cluster analysis of the MD simulations. The most populated N‐terminal conformation cluster is shown separately, and all other clusters are overlaid. Black arrows represent the position of the active site cavity, and the N‐terminus is shown in blue with the rest of the protein in rainbow.

We also performed SEC coupled small‐angle X‐ray scattering (SEC‐SAXS) on the full‐length and truncated Ct_EST and Mt‐EST at the Australian Synchrotron BioSAXS beamline (Table S4 and Figure 6). Rp_EST was excluded as the analytical SEC showed that it has multiple oligomeric states in solution (Table S3), which would confound the SEC‐SAXS observations.

The background‐subtracted scattering profiles confirmed that all Ct_EST and Mt_EST variants were monomeric in solution, based on molecular weight estimates obtained through Bayesian inference (Table S4). Both full‐length proteins displayed increased radii of gyration (*R*_g_) compared to their truncated counterparts (23.75 ± 0.12 vs. 20.89 ± 0.02 Å for Ct_EST, and 22.48 ± 0.03 vs. 18.83 ± 0.02 Å for Mt_EST). Notably, their maximal particle dimensions (*D*_max_) also increased in the full‐length proteins (90 and 83 Å) relative to the truncated forms (64 and 62 Å, respectively), suggesting that the presence of the N‐termini extends the protein structure in one dimension (Figure S8). Dimensionless Kratky plots showed a peak slightly beyond q*R*_g_ = √2 for all variants but did not return to baseline, as would be expected for fully compact proteins. This pattern indicates that both full‐length and truncated proteins retain some degree of conformational flexibility (Figure S8).

Because the AlphaFold3 models for both Ct_EST and Mt_EST yielded poor fits to the experimental SAXS profiles (χ^2^ = 3.90 and 20.72; CorMap *p*‐values = 1.75 × 10^−9^ and 5.76 × 10^−46^, respectively; Table S4), we used ensemble optimized modeling (EOM) to better account for conformational variability (Bernadó et al., 2007; Tria et al., 2015). EOM incorporated the AlphaFold3‐predicted structured core, while modeling the N‐terminal extensions (and C‐terminal tail for Ct_EST) as flexible regions. A random pool of 10,000 conformers was generated, from which a small subset was selected that best fit the SAXS data. The selected ensembles showed narrower, but multimodal *R*_g_ and *D*_max_ distributions compared to the random pool (Figure 6a), consistent with moderate conformational heterogeneity in the terminal regions (Figure 6b). Importantly, the ensemble models yielded improved fits to the experimental data (Figure 6b, c, Table S4), with lower χ^2^ values (2.42 for Ct_EST and 6.89 for Mt_EST) and better CorMap *p*‐values (1.45 × 10^−5^ and 7.63 × 10^−22^, respectively). Although not meeting the strictest criteria for a good fit (typically χ^2^ < 2 and CorMap *p* > 0.02) (Trewhella, 2022), the ensemble models represent a better agreement with the experimental data than the AlphaFold3 models.

Together, these results indicate that the N‐terminal regions of Ct_EST and Mt_EST adopt conformationally restricted ensembles in solution. This moderate flexibility is better captured by ensemble modeling than by single‐structure predictions.

For further investigation of the structure of the N‐terminus in these proteins, we also set up crystallization experiments using the full‐length constructs. However, we obtained only clusters of small crystalline needles and plates for Rp_EST and Ct_EST, respectively, and small single crystals that did not diffract for Mt_EST (Figure S9). We then set up screens with these full‐length proteins with limited proteolysis in the presence of trypsin and/or chymotrypsin, reasoning that any unstructured regions will be cleaved off. Under these conditions, Mt_EST produced more separate but still small single crystals, although they failed to diffract. By setting up screens with the truncated construct shMt_EST, we were able to obtain larger crystals, although still with poor diffraction. Ct_EST also produced single crystals under the limited proteolysis conditions, for which we were able to collect data; however, the poor quality precluded high‐quality structure determination. We did obtain crystals with shCt_EST, which allowed us to solve its structure to 1.9 Å (Table S5). The shCt_EST shows a clear α/β/α sandwich fold consistent with the SNGH hydrolase family. These findings suggest that removing the N‐terminal residues promotes crystal packing, where the N‐terminus seems to increase the disorder or dynamics of these proteins, consistent with the EOM models.

### 3.6 The N‐termini interact only transiently with the catalytic core in Ct_EST and Mt_EST

Our results suggest that in Rp_EST, the disordered N‐terminus promotes oligomerization, which may account for its increased thermal tolerance and loss of pNP‐butyrate hydrolysis activity compared to the truncated protein. However, this mechanism cannot explain the enhanced thermostability of Ct_EST and Mt_EST caused by the N‐terminus, which has no apparent effect on enzyme activity. To further investigate, we performed 1 μs MD simulations in triplicate for the full‐length (Ct_EST and Mt_EST) and truncated (shCt_EST and shMt_EST) proteins. Although this simulation length is not sufficient to observe folding transitions of intrinsically disordered regions, it can provide insight into their conformational behavior and dynamics.

The replicate simulations reached equilibration at different time points during the simulations but generally remained in the same state for over 200 ns once equilibrated (Figure S10). In both Ct_EST and shCt_EST, the presence of an extended disordered C‐terminus somewhat affected convergence, but stable analysis windows were still obtained.

We performed hierarchical clustering of the N‐terminal backbone conformations in the equilibrated regions of the simulations. Clustering was performed on every 10th frame of the equilibrated region of the simulations (Table S6), using the root mean square deviation (RMSD) of the N‐terminal backbone atoms (residues 1–12 for Mt_EST and 1–25 for Ct_EST), following alignment to the structured domain (residues 30 onwards). A shorter sequence length was chosen for Mt_EST as the rest of the N‐terminus remained mobile even when the first few residues adopted more stable positions. A range of RMSD cutoffs (2–6 Å) was evaluated, and a cutoff of 4 Å was selected as optimal for both proteins based on the cluster numbers, compactness, and interpretability (Table S6 and Figure S11). This resulted in 19 and 13 conformational clusters for Mt_EST and Ct_EST, respectively, with average intra‐cluster RMSD values below 3 Å.

For both proteins, the N‐terminus associated with the folded domain in the most populated cluster (38% for Ct_EST and 40% for Mt_EST; Figure 6d) but adopted a wider range of conformations and remained flexible in the remaining clusters. These conformations were generally short‐lived and diverse, with the most populated clusters only occurring in consecutive frames, suggesting that the N‐terminus did not adopt a stable secondary structure but instead sampled conformations within a partially restricted range. This dynamic behavior aligns with the flexibility inferred from the EOM analysis (Figure 6).

Comparison of per‐residue B‐factor profiles showed no significant differences in the dynamics of the catalytic domain between full‐length and truncated proteins (Figure S12), with larger fluctuations confined to terminal regions (Figure 6d). Notably, the N‐terminus did not associate with or affect the B‐factors at the active site of these proteins, in line with biochemical data showing that N‐terminal truncation does not affect pNP‐butyrate hydrolysis activity (Table 3).

These findings suggest that in Ct_EST and Mt_EST, the N‐terminal extension does not modulate the intrinsic flexibility of the folded core; instead, it adopts dynamic, transient interactions that are unlikely to impact the stability or activity of the catalytic domain directly. These may contribute to thermostabilization through mechanisms not captured by the presented biochemical assays or short‐timescale simulations. Further experimental and computational studies will be needed to resolve this mechanism in detail.

### 3.7 Increased thermal stability enhances polyesterase activity

Finally, we investigated whether the increased thermal stability of these enzymes correlates with improved polyesterase activity. Purified PpEST homologs were screened for activity at 40°C using coarsely ground PBAT, PET, and PBSA. This temperature was selected as all enzymes, except Pmt_EST, retained residual activity after heating at 45°C (Figure S2). Hydrolysis of PBAT and PET releases soluble terephthalic acid and related aromatic intermediates, which can be quantified by absorbance near 250 nm (Vongsouthi et al., 2025; Zhong‐Johnson et al., 2021). In contrast, PBSA degradation does not release any aromatic products, but like PBAT produces 1,4‐butanediol and derived alcohols. Hence, we developed an enzyme‐coupled assay that quantifies total alcohol released using equine alcohol dehydrogenase (EqAD) (Matos et al., 1985; Pietruszko et al., 1973), a broad‐specificity homodimeric enzyme that oxidizes alcohols while reducing NAD^+^ to NADH + H^+^, which can be monitored by using absorbance at 340 nm (Figure S13).

Initial assay validation confirmed EqAD activity with 1,4‐butanediol, producing a characteristic allosteric sigmoidal kinetic curve (Figure S13). We then applied this assay to 24‐h reactions containing 5 mg/mL PBAT with increasing concentrations of PpEST, and observed a correlation between enzyme concentration and both terephthalic acid production (absorbance at 250 nm) and alcohol release (EqAD activity). Notably, background PBAT hydrolysis without enzyme addition was also detected by both assays, with increased product formation with the enzyme.

**FIGURE 7:**
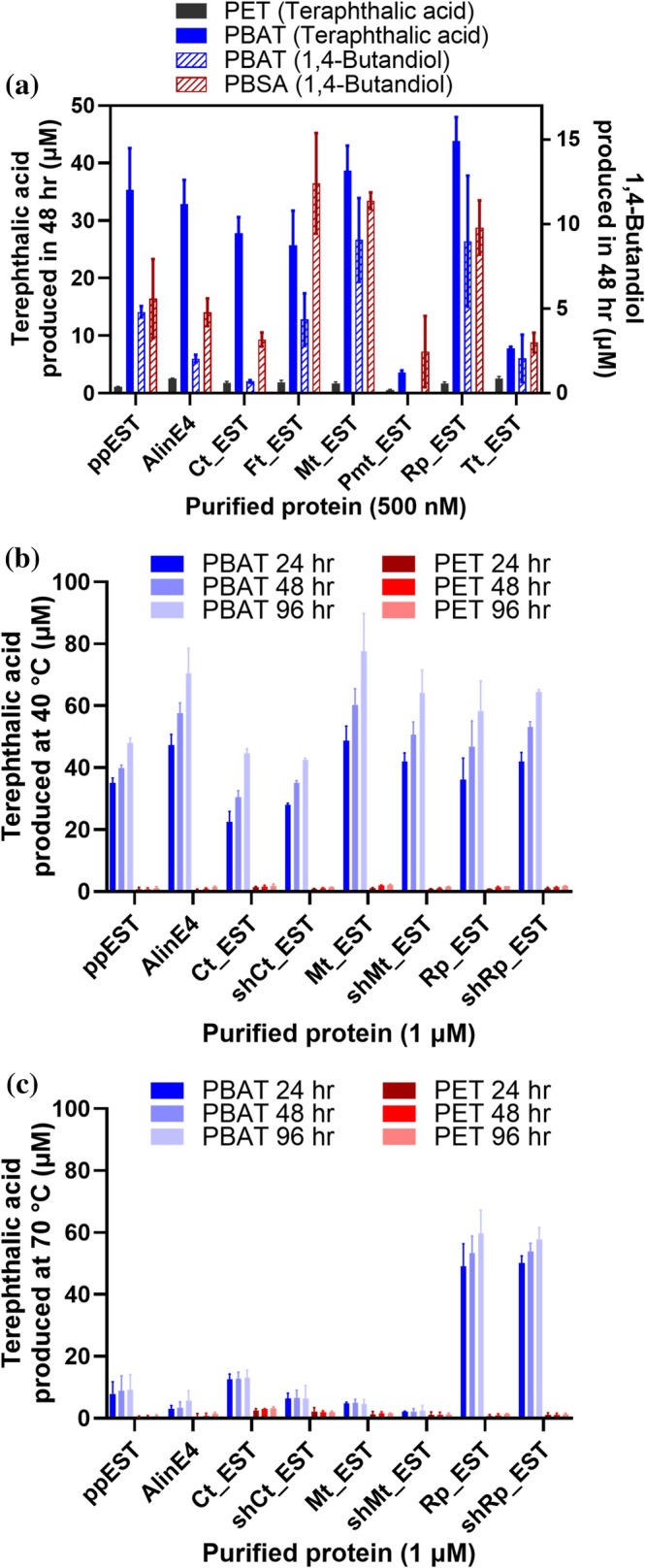
Polyesterase degradation activity by PpEST homologs. (a) Degradation of 5 mg/mL suspensions of PET, PBAT, and PBSA in 50 mM Tris (pH 8.0) at 40°C measured by detecting both terephthalic acid containing products with UV absorbance at 250 nm, and by detecting 1,4‐butanediol derived products by measuring alcohol dehydrogenase activity (*n* = 2). (b and c) Degradation activity of 5 mg/mL suspensions of PBAT and PET by the noted enzymes, measured over time by detecting terephthalic acid containing product release (*n* = 2), where reactions were set at 40°C for (b) and 70°C for (c).

Using this dual‐assay approach, all enzymes except Pmt_EST showed detectable activity against PBAT (Figure 7a). Pmt_EST exhibited only low activity with the absorbance assay and no activity with the EqAD assay. This is consistent with its negligible residual pNP‐butyrate hydrolysis after heating at 45°C, suggesting that the lower activity detected maybe because of its lower thermal stability. Interestingly, fewer 1,4‐butanediol‐containing products were detected from PBAT degradation compared to terephthalic acid‐containing products for all proteins, which could be a result of lower assay sensitivity. However, the change between the two detection methods is not uniform for all proteins, suggesting that there may be different reaction products formed from the different enzymes, for example, partially degraded polymers containing 1,4‐butanediol that are not as reactive with EqAD. All the proteins generated 1,4‐butanediol‐containing products from PBSA degradation, generally at similar or higher amounts compared to PBAT degradation. All the proteins also produced very small (almost negligible) amounts of terephthalic acid‐containing products from PET degradation.

To evaluate the effect of temperature and N‐terminal truncation, we performed time‐course hydrolysis of PBAT and PET at 40°C and 70°C for PpEST, AlinE4, Ct_EST, Mt_EST, Rp_EST, and their respective N‐terminal truncations (shCt_EST, shMt_EST, shRp_EST) (Figure 7b,c). A temperature of 70°C was selected as it was previously reported to be optimal for PET degradation by the highly thermostable WCCG variant of the Leaf Compost Cutinase (LCC) that has a *T*_m_ of 84.7°C (Akram et al., 2024; Tournier et al., 2020). Even at the elevated temperature of 70°C (Figure 7b, c), none of the tested enzymes demonstrated convincing terephthalic acid product from PET degradation, suggesting that these enzymes are either very inefficient at or unable to release terephthalic acid or derivatives from PET hydrolysis.

At 40°C, all enzymes showed a gradual increase in terephthalic acid derived products from PBAT over 96 h (Figure 7b). At this temperature, N‐terminal truncation had minimal impact on enzyme performance. At 70°C, while Rp_EST and shRp_EST (both with *T*_m_ >95°C) retained similar activity over 96 h as at 40°C, other enzymes showed only limited activity in the first 24 h, with no further product accumulation afterwards. This suggests some initial activity followed by heat inactivation. The similarity between Rp_EST and shRp_EST is consistent with 70°C being well below the *T*_m_ of both enzymes, indicating that both remain largely folded and catalytically competent under these conditions. Differences in stability between the two become apparent only at temperatures approaching or exceeding their unfolding transitions, as reflected when measuring residual activity over time at 99°C. Notably, Ct_EST and Mt_EST showed higher initial activity than their truncated forms, consistent with their improved thermostability. In addition, Ct_EST (*T*_m_ = 77.5°C) outperformed Mt_EST (*T*_m_ = 57.0°C), AlinE4 (*T*_m_ = 60.9°C), and PpEST (*T*_m_ = 59.6°C) in PBAT degradation at 70°C. Together, these results suggest that improved thermal stability in these enzymes improves their polyesterase activity at elevated temperatures. Additional investigation using quantitative methods such as mass spectroscopy or high‐performance liquid chromatography is required to identify the exact polyester degradation products produced in these reactions.

## 4 DISCUSSION

In this study, we have identified a distinct subgroup of bacterial polyesterases related to PpEST within the SGNH‐hydrolase superfamily, predominantly from marine‐associated Proteobacteria such as *Pseudomonas*, *Marinobacter*, and *Shewanella*, which are increasingly recognized for their plastic‐degrading capacity (Ruginescu & Purcarea, 2024; Wilkes et al., 2017). Among these, Rp_EST from *R. profundi* (a thermophile from deep‐sea vents; Marteinsson et al., 2010) showed the highest thermostability (*T*_m_ >99°C). A closely related enzyme from *R. marinus* (Rm_EST, 94% identity) could not be expressed with or without an extended N‐terminus, and further comparative studies may help elucidate sequence‐level determinants of protein expression in this family.

All homologs displayed esterase activity, with the highest activity observed for pNP‐butyrate, as previously reported for PpEST and AlinE4 (Li et al., 2020; Wallace et al., 2017). Notably, protein concentration strongly influenced apparent thermostability in our assays, consistent with prior findings that concentration, molecular crowding, and cellular constituents can impact protein unfolding and aggregation during thermal stress (Cheng et al., 2018; Speer et al., 2022).

The N‐termini of Ct_EST, Mt_EST, and Rp_EST conferred increased thermal stability, and both sequence and structural analysis suggest that they are likely intrinsically disordered regions (IDRs). Low AlphaFold3 confidence scores, disorder predictions, SAXS ensemble heterogeneity, and MD simulations all indicated highly dynamic conformations with only transient, non‐specific contacts with the catalytic core. This flexibility was further reflected in crystallization trials, where only truncated forms produced well‐ordered crystals, and in SAXS ensemble modeling, which captured their conformational range more effectively than single‐structure AlphaFold3 models. Sequence analysis revealed enrichment in disorder‐promoting residues (Van Der Lee et al., 2014), such as serine, threonine, proline, lysine, glutamate, and alanine, and a near‐complete absence of aromatic and bulky hydrophobic residues. Taken together, the sequence composition and structural behavior are consistent with classification as IDRs (Holehouse & Kragelund, 2023; Van Der Lee et al., 2014), resembling expanded self‐avoiding ensembles or “entropic bristles” often found in terminal disordered segments.

One potential way these N‐terminal IDRs contribute to stability is by promoting oligomerisation, which is a well‐known stabilizing mechanism that reduces entropy and aggregation under thermal stress (Álvarez‐Cao et al., 2018; Bjørk et al., 2004; Pucci & Rooman, 2017). In Rp_EST, the N‐terminus promotes oligomerisation, which likely contributes to its exceptional thermal tolerance (*T*₅₀ ~ 30 min at 99°C; *T*_m_ >99°C). PpEST also dimerised despite lacking an extended N‐terminus, indicating that the SGNH‐hydrolase family can employ at least two independent oligomerisation mechanisms. AlinE4, although monomeric in solution, showed comparable residual activity to Rp_EST and has been reported to form domain‐swapped dimers under different conditions (Li et al., 2020). However, its lower *T*_m_ of 61°C and lack of sustained PBAT degradation at 70°C suggest that residual activity may reflect refolding upon cooling, rather than true thermal stability at elevated temperatures. In contrast, Ct_EST and Mt_EST gained stability from their N‐terminal sequences without detectable oligomerisation. This indicates that while N‐terminus‐mediated oligomerisation can enhance stability in some enzymes in this family, other mechanisms must account for the stabilization observed in others, potentially arising from the disordered nature of these regions.

Only Rp_EST showed altered catalytic activity due to the N‐terminal region, where its truncated monomeric form exhibited ~2‐fold higher pNP‐butyrate activity. This loss could also be attributed to its oligomerization, which could reduce substrate access or alter catalytic dynamics, as has been observed in other systems (Deb et al., 2011; Simhadri et al., 2014).

Across all enzymes, trends in pNP‐ester hydrolysis did not consistently match PBAT degradation rates. For example, PpEST hydrolyzed pNP‐butyrate more slowly than Ct_EST but was more effective in PBAT degradation, implying different rate‐limiting steps for small‐molecule versus polymeric substrates. Factors such as substrate binding or polymer surface accessibility likely contribute to polymer hydrolysis efficiency and remain to be further investigated.

All enzymes degraded PBAT and PBSA, while PET degradation was minimal. Previous studies showed that PpEST can act on ionic phthalic polyesters composed of terephthalic acid and 1,2‐ethanediol (Haernvall et al., 2017). The full range of substrate specificities and degradation products remains to be explored in further work. Importantly, increased thermostability translated to sustained polyesterase activity at elevated temperatures. For example, Ct_EST outperformed Mt_EST, AlinE4, and PpEST in PBAT hydrolysis at 70°C, consistent with its higher *T*_m_ (77.5°C vs. 57°C–61°C), while Rp_EST retained robust activity at 70°C, in line with its exceptional thermal stability.

IDR‐mediated protein stabilization is distinct from conventional thermostabilizing strategies such as loop rigidification, hydrophobic core packing, or disulfide bond engineering (Chandler et al., 2020; Modarres et al., 2016). Unstructured or flexible termini are often destabilizing, as seen with N‐terminal His‐tags reducing thermal stability in several proteins (Booth et al., 2018). Similarly, a short, disordered N‐terminal extension in the FK506‐binding protein introduced a folding intermediate that modestly lowered stability despite limited structural change to the core (Korepanova et al., 2001). However, stabilizing effects have also been reported in a few cases. For instance, truncating a structured N‐terminus in a CE7 α/β‐hydrolase led to dramatic thermostability losses (Singh et al., 2017), and disordered N‐termini in small heat shock proteins enhance chaperone function under thermal stress (Zabci & Kocabiyik, 2024). In the enzymes presented in this work, dynamic N‐termini could be increasing thermal stability through IDR‐mediated mechanisms (Holehouse & Kragelund, 2023; Van Der Lee et al., 2014), such as buffering conformational fluctuations of the folded core, sterically shielding aggregation‐prone surfaces (entropic bristle effect), or modulating hydration layers around the protein. The exact mechanisms remain to be determined and warrant further investigation.

In conclusion, we have identified thermostable SGNH‐hydrolase family polyesterases with activity on PBAT and PBSA, with enzymes from thermophilic bacteria, notably Rp_EST from *R. profundi*, exhibiting exceptional thermal tolerance. We have demonstrated that increased thermostability enhances polyesterase activity at elevated temperatures, and that this stability can be modulated by unstructured N‐terminal extensions. These findings expand the mechanistic understanding of enzyme thermostability and inform future efforts to engineer robust enzymes for polyester degradation.

## AUTHOR CONTRIBUTIONS

**F. Hafna Ahmed:** Conceptualization; investigation; funding acquisition; writing – original draft; methodology; validation; visualization; writing – review and editing; formal analysis; project administration; data curation; supervision. **Lygie Esquirol:** Conceptualization; investigation; funding acquisition; writing – review and editing; visualization; validation; methodology; formal analysis; data curation; supervision. **Santana Royan:** Investigation; methodology; validation; visualization; writing – review and editing; formal analysis; data curation. **Mitchell M. Birgan:** Investigation; methodology; validation; visualization. **Nigel G. French:** Investigation; methodology; validation; visualization. **Sophia Newton:** Investigation; methodology; validation; visualization. **Alessandro T. Caputo:** Investigation; methodology; validation; visualization; writing – review and editing; data curation; supervision; formal analysis. **Colin Scott:** Conceptualization; funding acquisition; writing – review and editing; writing – original draft; formal analysis; supervision; project administration.

## CONFLICT OF INTEREST STATEMENT

The authors declare no competing interests.

## Supporting information


**Data S1:** Final set of 2123 sequences retrieved for ppEST like proteins used in this work is provided in the file “ppESTclade_jun24_99_nosignalseq_noduplicates.fasta.”

**Data S2:** Supporting information.

## Data Availability

The data that support the findings of this study are openly available in Protein Data Bank at https://www.rcsb.org/, reference number 9OTV.
