# Supplementary material for: Disordered N‐termini enhance the thermostability of SGNH‐hydrolase family polyesterases
Source: Protein Sci. 2025 Dec 22;35(1):e70402. doi: 10.1002/pro.70402 (PMC12720421; doi:10.1002/pro.70402)
Supplement: Supplementary file 2 — Data S2: Supporting information. [file PRO-35-e70402-s001.docx]

**Supplementary information for:**

**Disordered N-termini Enhance the Thermostability of
SGNH-Hydrolase Family Polyesterases**

F. Hafna Ahmed^a,b^*, Lygie Esquirol^a,b^, Santana Royan^c^, Mitchell Birgan^a,b^, Nigel G. French^a^, Sophia Newton^c^, Alessandro T. Caputo^c^, and Colin Scott^a,b,d,e^

^a^ Environment, CSIRO, Clunies Ross Street, Canberra, ACT 2601, Australia

^b^ Advanced Engineering Biology Future Science Platform, CSIRO, Canberra, ACT, 2601, Australia

^c^ Manufacturing, CSIRO, Clayton, VIC 3168, Australia

^d^ ARC Centre of Excellence in Synthetic Biology

^e^ School of Molecular Sciences, The University of Western Australia, Perth, WA 6009, Australia

**SI Data 1:** Final set of 2123 sequences retrieved for ppEST like proteins used in this work is provided in the file “ppESTclade_jun24_99_nosignalseq_noduplicates.fasta”

**Supplementary Methods**

**SDS PAGE Gels to assess protein expression**

To test protein expression, 500 µL from each culture was transferred to 1.5 ml microfuge tubes and spun down at 4,000 x g at 4 °C for 10 mins. The supernatant was discarded, and the resulting cell pellet was resuspended in 100 µL of lysis solution containing 50 mM Tris pH 8, 1x BugBuster Protein Extraction Reagent (Millipore) and ~33 nl DNAse I. The cells were left on ice to lyse for about 10 mins, and 5 µL from each sample was diluted with 15 µl of 50 mM Tris pH 8 and 5 µL of 4x NuPAGE^TM^ LDS Sample Buffer (Invitrogen) to make the whole cell protein fraction. The remaining lysed samples were spun down at 20,000 x g for 10 mins at 4 °C so that the supernatant contains the soluble protein fraction, 15 µL of which were mixed with 5 µL of 4x NuPAGE^TM^ LDS Sample Buffer (Invitrogen). All samples were heated at 90 °C for 3 mins and loaded onto pre-cast NuPAGE^TM^ 4-12 % Bis-Tris gels (Invitrogen). Gels were run for 30-40 min at 150 V in MES SDS running buffer (Invitrogen) and stained with AcquaStain Protein Gel Stain (Bulldog) for 30 mins, followed by destaining in water.

**Crystallography**

Crystals of the Ct_EST and shCt_EST variants were grown by vapor diffusion in MRC 2 lens sitting-drop plates (Swissci) setting up drops at a 1:1 ratio with 150 nl of protein and 150 nl of mother liquor using the Phoenix Crystal (Art Robbins) liquid handling platform. The shCt_EST construct was crystallized in 24% (w/v) PEG 10,000, 0.1 M Bis-Tris pH 5.7, 0.23 M ammonium sulfate at 293 K. Crystals grew after a couple of days and before flash cooling in liquid nitrogen, were cryoprotected with a final concentration of 15% glycerol added to the reservoir before overlaying onto the drop. Diffraction experiments were carried out at the Australian Synchrotron MX2 beamline ^1^, under nitrogen vapor at 100 K and a wavelength of 0.954 Å. Data reduction was carried out with XDS ^2^ for indexing and integration followed by scaling and merging with Aimless^3^. Automated implementation of the same programs was utilized with autoPROC^4^. Molecular replacement was carried out with Phaser^5^ via a model derived from AlphaFold2 ^6,7^. The model was iteratively built and refined using Coot 0.8.9 and autoBUSTER^8^.

**SI Table 1:** Coding sequences for the proteins expressed in this work. All sequences were cloned between the Nde1 and Xho1 restriction sites in the expression vector pET-29b(+) to introduce a C-terminal 6x Histidine tag (included in the sequence).

| **Protein** | **Coding sequence** |
| --- | --- |
| **Tt_EST** | atggctacaatactagtatttggagattcattaagcgcgggttatggtcttccgcctcagtccggctggccgtacctgctgcagcgtcaactggatgctttggagccgggtgcgcatcgtgttattgatgcgagcgttagcggtgagaccacggctggcggtgcgagccgtctcccaagattgcttgagcgccaccgtccggatgttgtggtcctggcattgggcgctaatgatggcctgcgtggcctgccgccgaaggcaatggaaactaacctggagcgcatgattctgatggcgaaacaatctgggtgcgaagttgtcttggttggtatgcgtctgccgcccaacctgggtccggactacaccgcggagttcgacgccgtgtatccgcgcttagccgcgcgctacccggttcatttcgtgccatttctgctggaggcgatcgccctggacccggacgcgtttcaagaagacggcctgcacccggtgctggaggcggtgccgaagctgagccagaccgtttggccggcaatcgcatcggctttggaagccgtgcgtaaacgtggtgcggcttccggccgtctcgagcaccaccaccaccaccactga |
| **Ft_EST** | atggggactgctccctcaggaggtacagcaggcgcgccagttattctggtgctgggcgatagcctgtcggcggcgtatggcatcgccctggacaaaggttgggtggccctccttcaagcacgtttgtctgcggcgggttacccgcatcgtgttgttaatgcgagcgttagcggtgagaccaccgcaggcggtctggcacgccttccggcattgttggatcgtcacaagccgcagtgggtcctggtggaactgggtggaaacgatggcctgcgtggtctgccgttgacccagctgcgtgacaacctgcgccgtatcgtggctctgtcccagaccgccggtgcgcgccctgttctgttcgagatgcgtattccgtccaactatggtccggactacgcggaaggttttcgtgcaagcttcgcgaccgtagcgagagaaacggcgaccccgttggtcccgtttttcttagccgcgattgctaccgatccgactgcgtttcaagaggacggcatccacccgacggctggcgcgcaggccgctatgctggacgctgtgtgggcaaccctgcaaccgctgttggagcgcctcgagcaccaccaccaccaccactga |
| **Pmt_EST** | atggggacagttctagtagtcggagattcaatctctgcggcattcggcttggacacggctcaaggttgggttgccctcctggagaagcgcctcgcggaacgtgatcgtgactaccaggtggtgaacgcaagcatttccggagacacctccgcgggtggcttggcacgtctgccggctttgttggacgctcaccgtccggaagtagtcatcatcgagctgggcggcaacgacggcctgcgtggtctgcctccggcgcagctgcagcagaatctggcgcgcatgattgatagcgcgcgtagcagaggcgcgcaagtgctgttgcttggcatgcgtctgccgccaaactatggtgaacgctacacccgtgcatttgaagcagttttcagcgatctggccgaggaaaaaggtgtggcgttagttccgtttttcctggagggtgttggtggcgtgccgggtatgatgcagccggatggtattcatccgaccgctgatgcccaaccgctgctgttggagcacgcgtggtcggcgctggagccgctgctgcgcctcgagcaccaccaccaccaccactga |
| **Ct_EST** | atgtcaaggactggaacagaagctcccgagaacctaccggtgctggcgccagccccgactgatgcggctccgacggcccgtccgcgtattgtcgtgctgggtgactccctgaccgcaggctttggtttgccacgtgaggcctcctatccgaccgttctgcagaaaaaactggatgcggcgggtctgaactaccaggtcatcaacgcgggaatcagcggtgataccagcgcaggcggtgtggaacgtttggactggtctttggacggcgacgttcgtattgttatcctggctctgggcgccaacgatggcctgcgtggtttgccgttaacccagatggaagcaaatctgcgcaccattatcgagcgcgcgagagcacgtggtgcgcaggttatcctcgcgggtctgaaggcaccggctgaagcgggccctgactacggtgctcagttcgaggcggtttatcgtaagctggcccagcaataccgcctgccgctgatcccgagccttctggagggtgtggctgggcgtgaggaactgaaccaagaagacggcattcacccgaatgcgcgtggcgctgcgattgtagctgataacgtgtggaaggtgttggagccggttgcgcgccaacaactggcccagtcgcaaagcaatccggatgctccgcctgcggcaaaaccggcgggtgcgcgcctcgagcaccaccaccaccaccactga |
| **shCt_EST** | atgccgcgtattgtcgtgctgggtgactccctgaccgcaggctttggtttgccacgtgaggcctcctatccgaccgttctgcagaaaaaactggatgcggcgggtctgaactaccaggtcatcaacgcgggaatcagcggtgataccagcgcaggcggtgtggaacgtttggactggtctttggacggcgacgttcgtattgttatcctggctctgggcgccaacgatggcctgcgtggtttgccgttaacccagatggaagcaaatctgcgcaccattatcgagcgcgcgagagcacgtggtgcgcaggttatcctcgcgggtctgaaggcaccggctgaagcgggccctgactacggtgctcagttcgaggcggtttatcgtaagctggcccagcaataccgcctgccgctgatcccgagccttctggagggtgtggctgggcgtgaggaactgaaccaagaagacggcattcacccgaatgcgcgtggcgctgcgattgtagctgataacgtgtggaaggtgttggagccggttgcgcgccaacaactggcccagtcgcaaagcaatccggatgctccgcctgcggcaaaaccggcgggtgcgcgcctcgagcaccaccaccaccaccactga |
| **St_EST** | atgcagtcagcactacaagcgcccgctgtagagttagctcgtgcggcgctgccggaggtgctgaccccggacccgcgtgtagaccgctcgggttggcctgtcatcgttgcgttcggcgacagcctgactgcgggcttgggcgtggacccggaccgcaactacccgagccagctgcaagcgctgttggatgaacgtggttaccgctatagagtggttaatgcaggcgtcagcggtgaactgaccgctggcggtctgagccgtgtggaccaggttttggagcaccgtccggcagttgtcattctggagctgggtgcgaacgatggtatgcaggcccgtccggttgcggaggtgcgcgaaaacctggcggggatcattgcacgtctacaagaagaaggcgttgccgttctgctcgctggcatgcgcgcgccaccaaatcatggtccggagtacgaagctgcgttcgcccaagtgtatccggatctggctgcggaatttggtgtgccgcttatcccgtttttcctggagggtgtcgccggctccccgtatctgaaccagcgtgatggcatccacccgacggcgcagggttacgcaattgtggtgcgtaccgttttgtctgcactggagccgttgctgcaaccgcctctcgagcaccaccaccaccaccactga |
| **Rb_EST** | atgcaagatagtgtttcacccgtaagggtcgtgttcctgggcaactctttaacctctggttatggtctgtcgatcaaagaggcgtacccggctttgctgcaaacgcgtgttgatagcctgggttggcctgtagttatggttaacgccggtgttagcggtgatacctccagcggtggcttgagacgtttgggctggcagctgcgtatgccaccggacgttctgatcgtggccctcggcggtaatgatggcttgcgcggtatcctgccggcgctgactaagagcaatctgattcagattgtgaaccaaaccaaggctgcgaactccgaagcgaccattatcgtggcgggtatgcagatgccgccgaatcttggcgcacagtttcaggaggagttccgcgcagtcttcccggcggtggcggaagagacgggtgcggcgctgatcccgtttctgttggagggtatcggcgggatcgccaaactgaaccaaccggacggcatccatccgaccgcagctggccagcgtattctggcggacaacgtgtgggaaattctcgcaccggttctgcaaagccacattgaacgtctcgagcaccaccaccaccaccactga |
| **Rm_EST** | atgggaggggctccagaacctcccaggtcagaggcaagcccggacaccaccgcccctgcggtgcgcaacaccgccagagctgaacgcaccattaacgtcctcgtcttgggaaacagcctggcggctggctatggcttgtccccggacgaggcctttccggcagttctgcaacgtaaggtggattcgctgggttggccagttcgtattatcaacgcgggcctgtctggtgaaacgagcgcaggtggtttgcgtcgcatcgaatggctgctgagagaacgtatcgatgttctgatcctggaactgggcgcgaatgatggtttgcgtggtattgacccggaggtgacccgtcgtaatttacaaggtatcatcgataaagttcgtgcgcgttatccggacgctgatattatcctggctggcatgcagctaccaccgaatctgggtccggactacaccgccgcgttccgtgcgatttacccggaactggctcgcgcgaacgatgcgcacctgattccgttcctgttggaaggcgttggtggcgtgccggagttgaatcaggcagacggcattcatccgactgcggagggtcagcgcatcgtggcagagaacgtgtggcgcgttctgcgtccggtacttgagcgtcaactgagccgtccgacgcgcaacgcgtccctcgagcaccaccaccaccaccactga |
| **shRm_EST** | atgattaacgtcctcgtcttgggaaacagcctggcggctggctatggcttgtccccggacgaggcctttccggcagttctgcaacgtaaggtggattcgctgggttggccagttcgtattatcaacgcgggcctgtctggtgaaacgagcgcaggtggtttgcgtcgcatcgaatggctgctgagagaacgtatcgatgttctgatcctggaactgggcgcgaatgatggtttgcgtggtattgacccggaggtgacccgtcgtaatttacaaggtatcatcgataaagttcgtgcgcgttatccggacgctgatattatcctggctggcatgcagctaccaccgaatctgggtccggactacaccgccgcgttccgtgcgatttacccggaactggctcgcgcgaacgatgcgcacctgattccgttcctgttggaaggcgttggtggcgtgccggagttgaatcaggcagacggcattcatccgactgcggagggtcagcgcatcgtggcagagaacgtgtggcgcgttctgcgtccggtacttgagcgtcaactgagccgtccgacgcgcaacgcgtccctcgagcaccaccaccaccaccactga |
| **Rp_EST** | atgcaagatacatcaaaaagttctgctagctccagcgaaggcagcccggataccacggctaaaaccccgacgcatgcagctcgtgcggcacagccgattaccgttttggtcttgggaaacagcctggctgccggttatggtctctcgccggatgaagcgttcccggcgattctgcaacgtaaggtggattccctgggctggcctgtgcgtatcctgaacgccggcctgtctggtgaaaccagcgcggggggtctgagacgtattgactggctgctgcgcgaacgtatcgacgtactgattctggaattaggcgcaaacgatggtcttcgcggtattgacccggaggtgacccgtcagaacttacaaggtattatcgataaagttcgtgcgcgttacccggaggccgacatcatcttggcgggtatgcagctgccaccgaatctgggtccggcatacaccgctgcgttccgcgcgctgtatccagagctcgcgcgcgcgaacaacgcatacctgatcccgtttctgctggagggcgtcggtggtgttccggagttgaatcaggcagacggcatccacccgactgccgagggccaacgcatcgtggcggaaaatgtttggcgtgtgttgcgtccggttttggagcgccgtctgggcttggcgctcgagcaccaccaccaccaccactga |
| **shRp_EST** | atgattaccgttttggtcttgggaaacagcctggctgccggttatggtctctcgccggatgaagcgttcccggcgattctgcaacgtaaggtggattccctgggctggcctgtgcgtatcctgaacgccggcctgtctggtgaaaccagcgcggggggtctgagacgtattgactggctgctgcgcgaacgtatcgacgtactgattctggaattaggcgcaaacgatggtcttcgcggtattgacccggaggtgacccgtcagaacttacaaggtattatcgataaagttcgtgcgcgttacccggaggccgacatcatcttggcgggtatgcagctgccaccgaatctgggtccggcatacaccgctgcgttccgcgcgctgtatccagagctcgcgcgcgcgaacaacgcatacctgatcccgtttctgctggagggcgtcggtggtgttccggagttgaatcaggcagacggcatccacccgactgccgagggccaacgcatcgtggcggaaaatgtttggcgtgtgttgcgtccggttttggagcgccgtctgggcttggcgctcgagcaccaccaccaccaccactga |
| **Pzt_EST** | atgggagaagggtcaaaaaaggaggctagtaaggcgagcgaagaggctccgcgtgatccgaagaccgaagttaaagacaatgcaaaagttattctgtttttcggaaactctctgaccgcaggctatggtattgatatggaagaggcgtttccggcactgatccaggcgcgtctggacagcttaggcctgaactacaaggccattaattcgggcttgtctggcgaaactaccagcagcggtctgaacagactgaaatgggtcctcgatcaacaagttgacatcttcgtgttagagttgggcgcgaatgatggtctgcgcggtgtttccctggtagaaacgaaaaagaacctgcagagcatgatcgacttggtgcgtgctaaatccccggatattaaaatcatcctggcgggtatgcagattccgcctaatatgggtcaggcgtacgcgattgagtttggtcgtttgttcccggaacttgccgaagcgaacaacgtggacctgatcccgttcctgctggagaacgtggccggccgcgcagagttgaacattgaggacggcatccatccgaccgctgaaggtcacaagatcgtgatggaaaatgtttggcaggtcttggaggagcacctggttgcgccaaagctggaccaactcgagcaccaccaccaccaccactga |
| **Mt_EST** | atgggagaatcaaaaaagcagaggcaaacagacaacaccaccgaaagcaccgttgaagagaacaaggaggaaaagcagacccagagcaaagtcatcctgttcttcggcaactcgctgaccgcggcatacggccttgagacagaggatggttttccgcaccgtattcaactgcgcctggacagcttaggtcttgactacaaagtgatcaattctggcctgtccggtgaaactacgagcggcggtttgaatcgtctagactgggttttgaaccagaagatcgacatctttgtactggagctgggtgccaatgatggcctgcgcggcattccgctgtctgagaccaaagaaaacctgcaaaagatcatcgataaagttcgtgaaaagaacaacgaaaccacgattctgttagcgggtatgcagattcctccgaatatgggtcaagattataccactgagttcaaaagcatgtttccggatctggcgaaaaagaataacgttctcctgattccgttcttgctggagaatgttgcaggtatcccagaattgaacctggaggacggcattcatccgaccgctgaaggtcaaaagatcgtggcgaacaacgtgtggaaagtgttggagccggtcgtgtccccgaaactcgagcaccaccaccaccaccactga |
| **shMt_EST** | atgaaagtcatcctgttcttcggcaactcgctgaccgcggcatacggccttgagacagaggatggttttccgcaccgtattcaactgcgcctggacagcttaggtcttgactacaaagtgatcaattctggcctgtccggtgaaactacgagcggcggtttgaatcgtctagactgggttttgaaccagaagatcgacatctttgtactggagctgggtgccaatgatggcctgcgcggcattccgctgtctgagaccaaagaaaacctgcaaaagatcatcgataaagttcgtgaaaagaacaacgaaaccacgattctgttagcgggtatgcagattcctccgaatatgggtcaagattataccactgagttcaaaagcatgtttccggatctggcgaaaaagaataacgttctcctgattccgttcttgctggagaatgttgcaggtatcccagaattgaacctggaggacggcattcatccgaccgctgaaggtcaaaagatcgtggcgaacaacgtgtggaaagtgttggagccggtcgtgtccccgaaactcgagcaccaccaccaccaccactga |
| **AlinE4** | atgggagaatcaagggtaatactagcttttggcgacagcctgtttgcaggttatggcctcgacaagggtgaaagctatccggcgaagttggagacggccctgagaagccacggcatcaacgctcgtatcattaacgctggcgtgtccggtgataccactgcggcaggcctgcaacgtatcaaattcgtgctggattctcaaccggacaaaccggaattggcgattgtcgagcttggcggtaatgacctgttacgtggtctgtcgccggcggaagcgcgccagaatctgtccggcatcctggaggagctgcagcgccgtaaaatcccgattttgttaatgggtatgcgtgcgccaccgaaccttggtgcaaagtaccaacgcgagttcgatggtatctatccttacctggccgaaaaatacgatgctaagctggttccgtttttcctggaggctgtggcggatcgtccggacctgattcagaaagaccacgttcatccgaccgcgcgtggtgttgaagaattggttagcgcaaccagcaacgccgtggccaaggcgctgccggcgaagaaactcgagcaccaccaccaccaccactga |
| **PpEST** | atgatgggtacgctgcttgtggtaggtgacagcatcagcgccgcatttggtttggattcacgtcaggggtgggtggcacttctggaaaaacgtctctcggaagaaggctttgaacacagtgttgtcaatgcaagcatttctggtgatacgagtgcgggcggcgcagcccgtctgtctgcactgctggccgaacataaaccggagctcgtaattattgagctgggcggtaatgacggcctgcggggccagcctccggcccagctgcaacagaatctggcgtcgatggtggagcagtcccagcaggccggcgctaaagtgttactgctgggtatgaagctgcccccgaattatggcgtgcgttacaccaccgcatttgcgcaggtttttaccgatttggccgaacaaaaacaagtgagcctggtcccattttttctggagggtgtgggtggtgttccgggaatgatgcaggccgacgggatccatccagcagaggcagcccaggaaatcttgctggataacgtttggccgaccttaaagccgatgcttctcgagcaccaccaccaccaccactga |


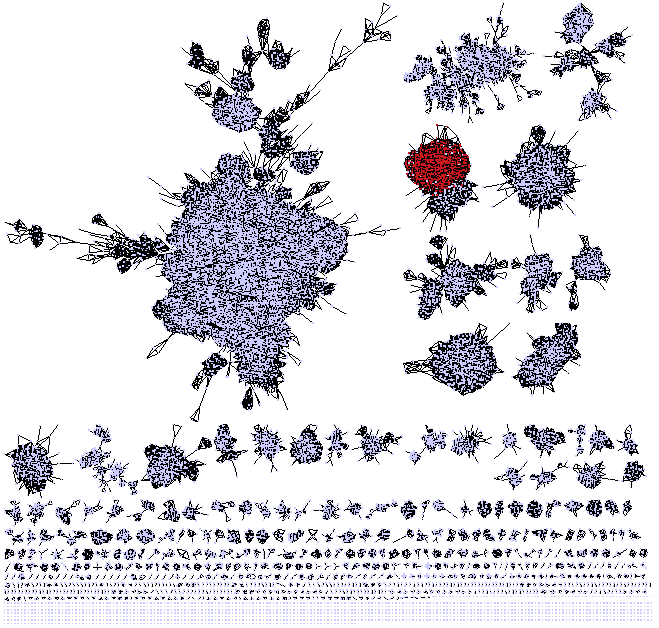

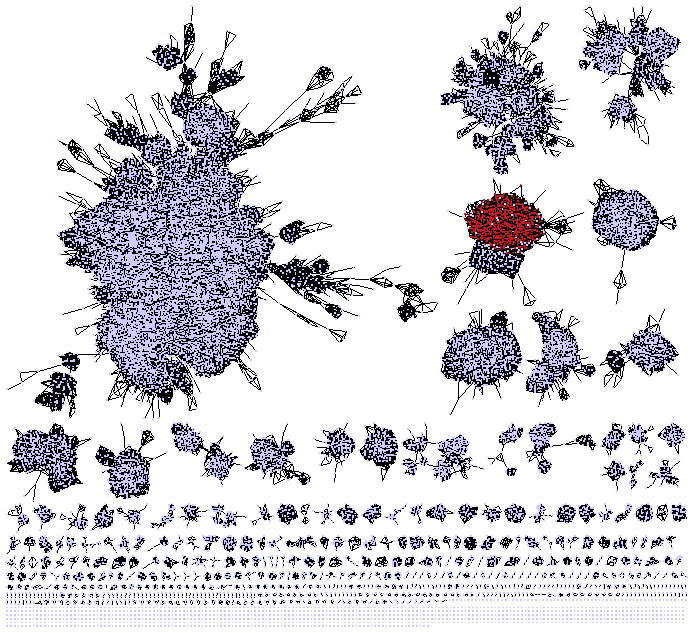


**SI Figure 1:** Clustering of PpEST homologues (red) in the same sequence similarity network (SSN) shown in Figure 1 panels A and B, at alignment score cut-offs of 27 (left) and 25 (right).

**SI Figure 2:** Initial characterisation of the thermostability of PpEST homologues. **A.** Residual activity with pNP-butyrate by cell cultures expressing ppEST homologues. Samples were heated at different temperatures for 10 min and cooled on ice for another 10 min. **B.** Residual activity with pNP-butyrate by purified ppEST homologues after heating at different temperatures for 10 min and cooling on ice for another 10 min.

**
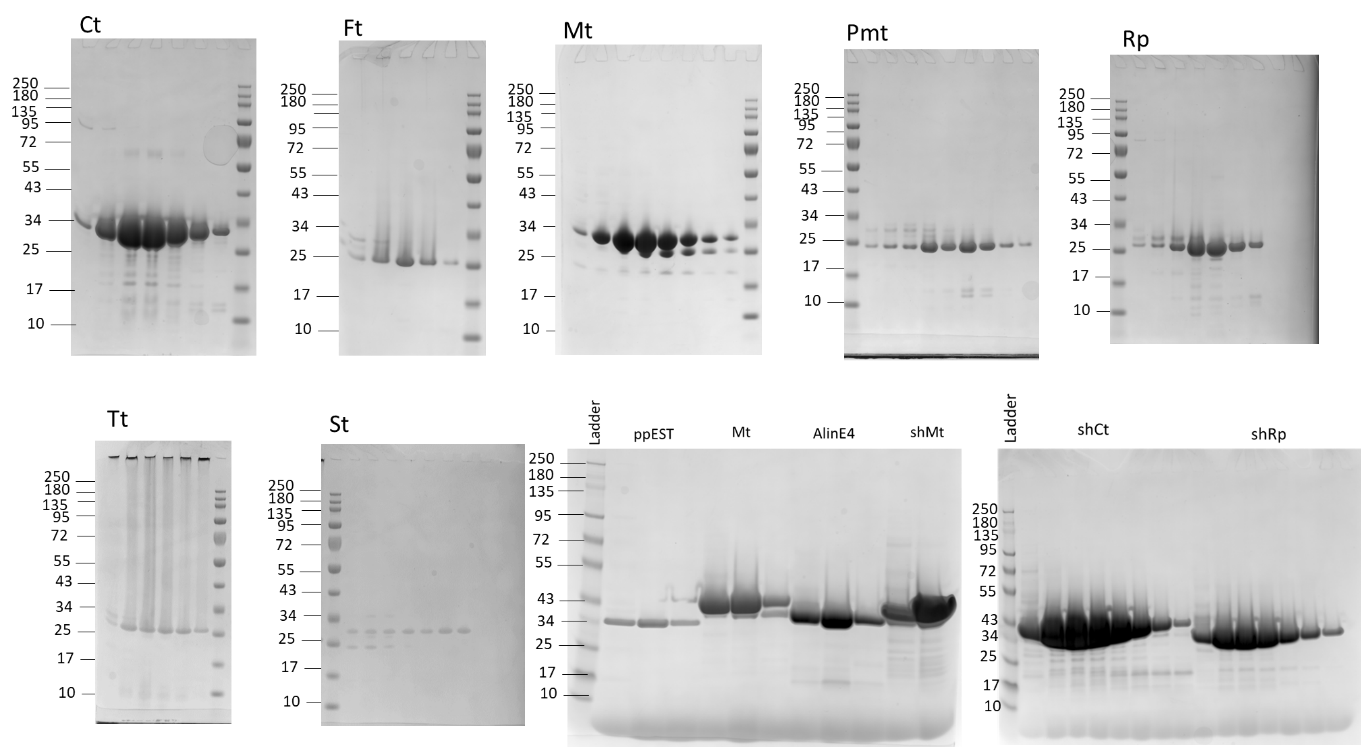
**

**SI Figure 3.** Protein gels after size exclusion chromatography for all proteins purified in this work.


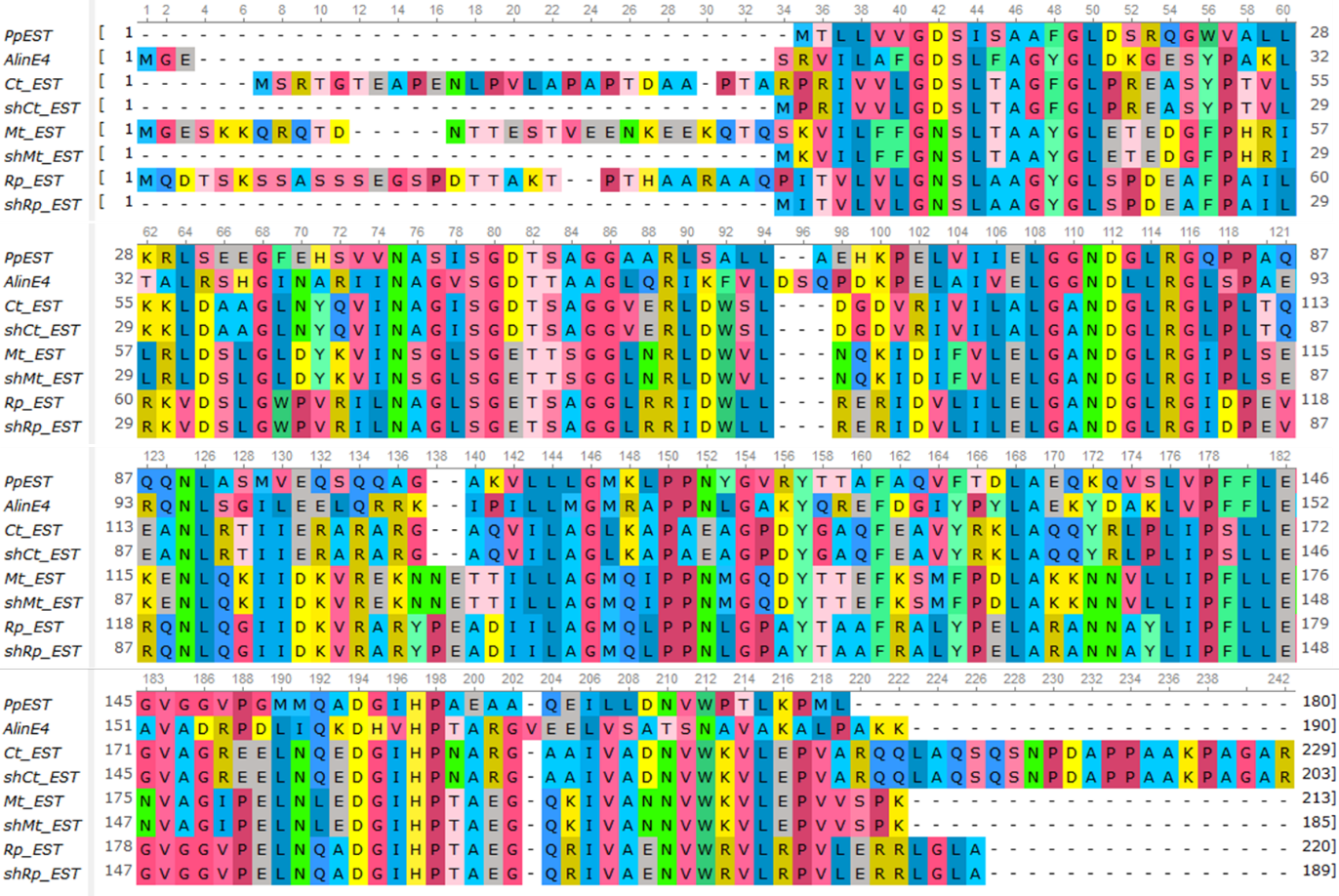


**SI Figure 4**. Sequence alignment of PpEST, AlinE4, Ct_EST, shCt_EST, Mt_EST, shMt_EST, Rp_EST and shRp_EST.


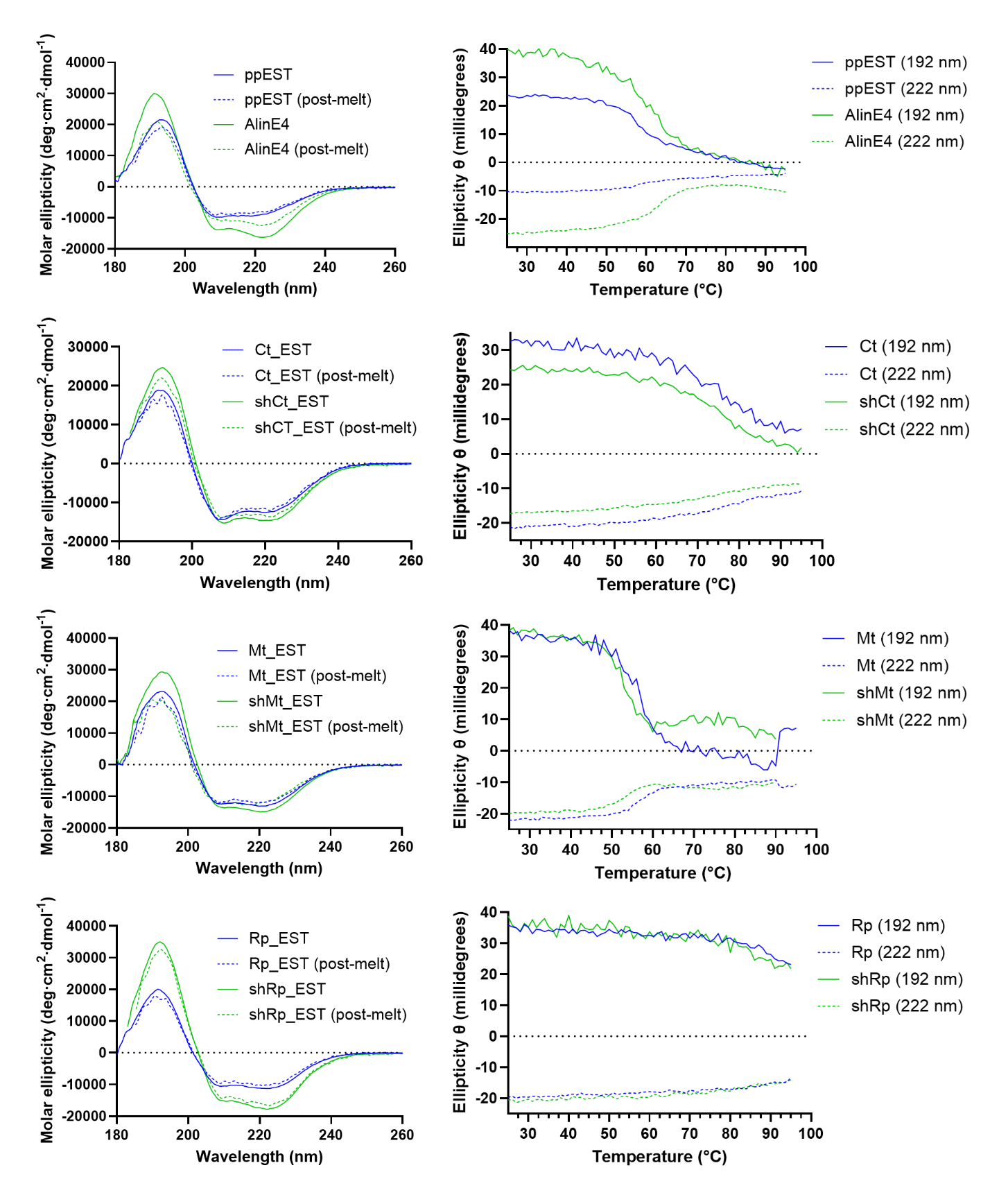


**SI Figure 5:** CD spectra comparing the full-length and short proteins. Left-hand side: Spectra before and post melting of the same sample. Right-hand side: The melting curves extracted for both 195 nm and 222 nm are shown for single experiments.

**SI Table 2.** Secondary structure composition of the proteins from CD-spectroscopy calculated by BestSel

|  | Ct | shCt | Mt | shMt | Rp | shRp |
| --- | --- | --- | --- | --- | --- | --- |
| Helix (%) | 31.6 | 29.9 | 29.8 | 33.8 | 25.7 | 35.5 |
| Antiparallel (%) | 10.7 | 15.9 | 6 | 5.9 | 13.2 | 5.8 |
| Parallel (%) | 2 | 1.4 | 8.6 | 8 | 3.7 | 9.5 |
| Turn (%) | 14.8 | 15 | 10 | 8 | 13.4 | 14.8 |
| Others (%) | 40.9 | 37.8 | 45.6 | 44.3 | 44 | 34.4 |

**SI Table 3.** Summary of analytical size exclusion chromatography of purified proteins (Cytiva Superdex 200 Increase 10/300 GL). MW was calculated by plotting Kav against Log(MW) for the standard, where Kav = (Ve-Vo)/(Vc-Vo) (Ve – elution volume, Vo – void volume, Vc – column volume).

|  | **calculated MW from SEC (kDa)** | **theoretical MW (kDa)** | **Predicted oligomerisation** |
| --- | --- | --- | --- |
| **With extra N-terminal sequences** | | | |
| Mt_EST | 33.9 | 24.8 | Monomer |
| Ct_EST | 34.8 | 25.1 | Monomer |
| Rp_EST-peak1 | 1173 | 24.6 |  |
| Rp_EST-peak2 | 121 | 24.6 | Tetramer |
| Rp_EST-peak3 | 85.3 | 24.6 | Dimer |
| Rp_EST-peak4 | 25.8 | 24.6 | Monomer |
| **Without extra N-terminal sequences** | | | |
| shCt_EST | 23.7 | 22.5 | monomer |
| shMt_EST | 20.9 | 21.5 | monomer |
| shRp_EST | 19.0 | 21.6 | monomer |
| AlinE4 | 18.4 | 21.7 | Monomer |
| PpEST-peak1 | 53.7 | 20.2 | Dimer |
| ppEST-peak2 | 17.0 | 20.2 | Monomer |
| **Standards (BioRad Gel Filtration Standard #1511901)** | | | |
| Thyroglobulin | 676 | 670 | n/a |
| Bovine γ-globulin | 154 | 158 | n/a |
| Chicken ovalbumin | 45.3 | 44 | n/a |
| Equine myoglobin | 16.8 | 17 | n/a |
| Vit B12 | 1.35 | 1.35 | n/a |
| **Control** |  |  |  |
| AmilCP | 49.2 | 55 | monomer |

**
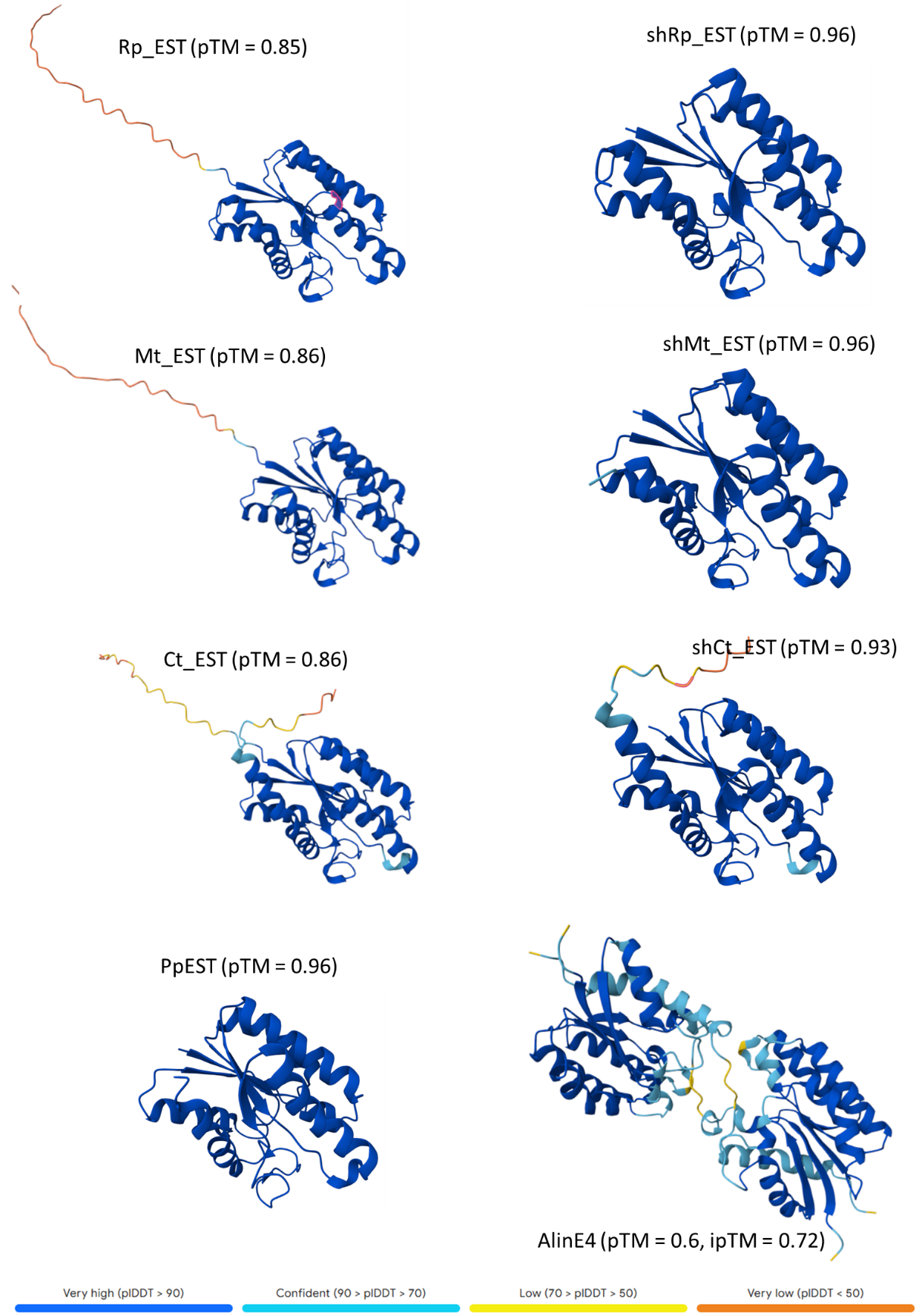
**

**SI Figure 6:** AlphaFold3 models of Ct_EST, Mt_EST, Rp_EST, shCt_EST, shMt_EST, shRp_EST, ppEST and AlinE4. The predicted template modeling (pTM) score and the interface predicted template modeling (ipTM) scores are provided, where a pTM score above 0.5 means prediction may be similar to the true structure. The ipTM score measures the accuracy of the predicted complex relative to the positioning of the subunits, where values higher than 0.8 represent high-quality predictions, and values between 0.6 and 0.8 suggest predictions could be correct or incorrect.

**
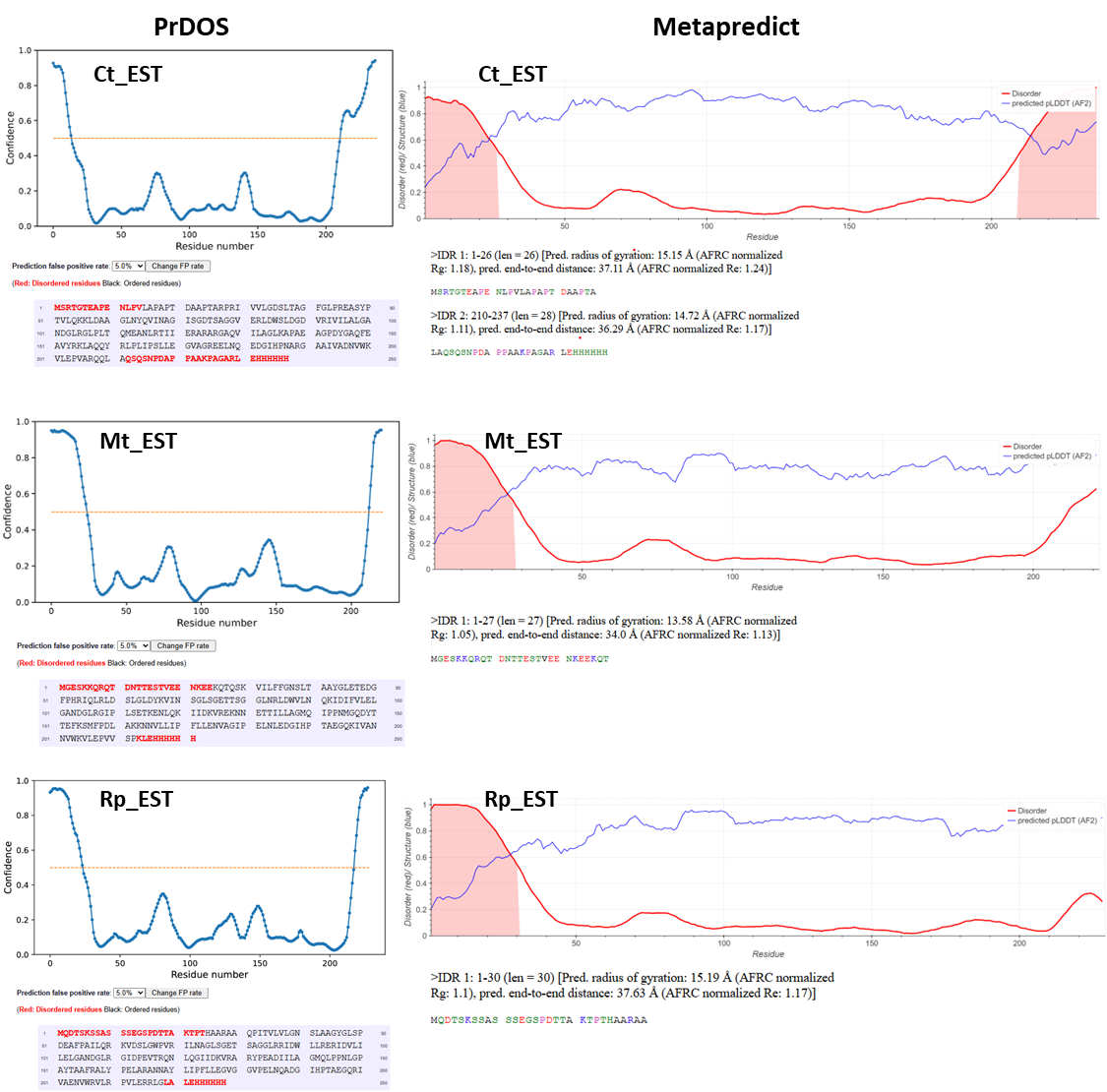
**

**SI Figure 7:** Intrinsically disordered region prediction for Ct_EST, Mt_EST and Rp_EST using PrDOS^9^ and Metapredict^10^. Predicted intrinsically disordered residues (excluding the HIS-tag) were: Ct_EST – 1-14 and 212-231 (PrDOS), 1-26 and 210-231 (Metapredict), Mt_EST – 1-24 and 213-215 (PrDOS), 1-27 (Metapredict), Rp_EST – 1-24 and 219-222 (PrDOS), 1-30 (Metapredict).

**SI Table 4.** SAS sample details, data collection, analysis, and 3D modelling details for biomolecules in solution.

| (*a*) Sample details | | | | |
| --- | --- | --- | --- | --- |
| Organism | *Chloracidobacterium thermophilum* (WP_014100090.1) | | *Maribacter thermophilus* (WP_047245951.1) | |
| Source (Catalogue No. or reference) | Expressed and purified from *E. coli* NEB T7 Express cells | | | |
| Samples | Ct_EST | shCt_EST | Mt_EST | shMt_EST |
| *Scattering particle composition* | | | | |
| Protein(s) | UniProtKB ID G2LJ66, residues 28-255 | UniProtKB ID G2LJ66, residues 54-255 | UniPARC ID UPI0006411730, residues 20-231 | UniPARC ID UPI0006411730, residues 48-231 |
| DNA/RNA(s) | - | - | - | - |
| Carbohydrates/glycans | - | - | - | - |
| Stoichiometry of components | 1 | 1 | 1 | 1 |
| *Sample environment/configuration* | | | | |
| Solvent composition | 25 mM Tris-HCl pH 7.6, 150 mM NaCl, 5% (v/v) glycerol | | | |
| Sample temperature (°C) | 25 | | | |
| In beam sample cell | 1 mm quartz capillary with co-flow | | | |
| *Size Exclusion Chromatography SEC-SAS* | | | | |
| Sample injection concentration, mg/m | 10.10 | 10.15 | 9.95 | 10.18 |
| Sample injection volume, mL | 0.5 | 0.5 | 0.5 | 0.5 |
| SEC column type | Superdex S200 5/150 GL (Cytiva) | | | |
| SEC flowrate, mL/min | 0.4 | 0.4 | 0.4 | 0.4 |
| (*b*) SAS data collection | | | | |
| Data acquisition/reduction software | Data was reduced by Fast Azimuthal Integration using Python (PyFAI) with customized algorithms written for the BioSAXS beamline | | | |
| Source/instrument description or reference | The BioSAXS beamline with Dectris Pilatus3 X 2M detector at the Australian Synchrotron, part of ANSTO (Tan *et al.*, 2025) | | | |
| Wavelength (Å) | 1.00 (12.4 keV) | | | |
| Measured *q*-range (*q_min_* – *q_max_*; Å^-1^) | 0.0068-0.4748 | | | |
| Method for scaling intensities | Absolute scaling referenced to water | | | |
| Exposure times | 1 s exposures per frame | | | |
| Final number of sample frames used for averaging. | 25 | 19 | 22 | 23 |
| (*c*) SAS-derived structural parameters | | | | |
| Methods/Software | BioXTAS RAW (RAW 2.3.0, Hopkins et al 2024), PRIMUS/qt (ATSAS 3.3.0; Manalastas-Cantos et al., 2021), GNOM (Svergun 1992) and AUTORG (Petoukhov et al. 2007) | | | |
| *Guinier Analysis* | Ct_EST | shCt_EST | Mt_EST | shMt_EST |
| *I*(0) ± σ (cm^-1;^ a.u) | 2.874E-03±9.591E-06 | 9.53E-03±6.997E-06 | 8.949E-03±8.207E-06 | 7.886E-03±6.146E-06 |
| *R*_g_ ± σ (Å) | 23.7536±0.1155 | 20.8909±0.0241 | 22.4778±0.032 | 18.8287±0.0234 |
| *min < qR_g_* < *max* limit (or data point range) | 0.2271-1.3289 | 0.0241-1.2960 | 0.1518-1.2997 | 0.1801-1.3181 |
| Linear fit assessment (r^2^) | 0.9322 | 0.9441 | 0.9425 | 0.9819 |
| *PDDF/P(r) analysis* | Ct_EST | shCt_EST | Mt_EST | shMt_EST |
| *I*(0) ± σ (cm^-1;^ a.u.) | 2.87E-03±8.42E-06 | 9.36E-03±5.24E-06 | 8.85E-03±6.99E-06 | 0.00786±6.34E-06 |
| *R*_g_  ± σ (Å) | 24.32±0.0985 | 20.34±0.0130 | 22.34±0.0168 | 18.76±0.0199 |
| *d*_max_ (Å) | 90 | 64 | 83 | 62 |
| *q*-range (Å^-1^) | 0.0096-0.4748 | 0.0068-0.4748 | 0.0068-0.4748 | 0.0096-0.4748 |
| *P*(*r*) fit assessment (χ^2^) | 2.5336 | 6.9115 | 6.7819 | 3.6954 |
| (*d*) Scattering particle size | | | |  |
| Methods/Software | BioXTAS RAW (RAW 2.3.0, Hopkins et al. 2024) & PRIMUS/qt (ATSAS 3.3.0; Manalastas-Cantos et al. 2021) for M from Bayesian inference (Hajizadeh et al., 2018), volume of correlation Vc (Rambo & Tainer, 2013), corrected Porod volume (Piiadov et al. 2019) | | | |
|  | Ct_EST | shCt_EST | Mt_EST | shMt_EST |
| *Volume estimates* | | | | |
| Porod volume, *V_p_* (Å^3^) | 6.01E04 | 4.01E04 | 4.99E04 | 3.41E04 |
| *Molecular weight (M) estimates (kDa)* | | | | |
| From chemical composition | 25.11 | 22.53 | 24.76 | 21.54 |
| From SAS, concentration independent method (Bayesian Inference with Probability; Confidence Interval with Probability) | 28.9 (68.4%), 27.9-29.9 (93.3%) | 19.9 (51.8%), 19.0-21.5 (94.3%) | 24.3 (41.7%), 22.8-25.2 (96.5%) | 18.7 (62.1%), 17.8-19.6 (94.0%) |
| From SAS, concentration independent method (Volume of correlation) | 26.1 | 18.8 | 22.8 | 17.0 |
| Vc (Å^2^) | 276.1 | 219.7 | 251.1 | 198.6 |
| Qr (Å^3^) | 3.21E03 | 2.31E03 | 2.80E03 | 2.10E3 |
| From SAS, concentration independent method (Porod Volume) | 30.6 | 21.0 | 25.5 | 19.5 |
| Corrected Vp (Å^3^) | 3.69E04 | 2.53E04 | 3.08E04 | 2.34E04 |
| (*e*) Modelling (a complete sub-panel for each method) | | | |  |
| *Shape modelling method(s)* | Dummy-atom modelling and subsequent averaging (Manalastas-Cantos et al., 2021, Svergun 1999, Volkov & Svergun 2003) | | | |
|  | Ct_EST | shCt_EST | Mt_EST | shMt_EST |
| Software | DAMMIN & DAMAVER | | | |
| *q-*range for fit (*q_min_* – *q_max_*; Å^-1^, nm^-1^) | 0.0096-0.4748 | 0.0068-0.4748 | 0.0068-0.4748 | 0.0096-0.4748 |
| Symmetry/anisotropy assumptions | P1/unknown | P1/unknown | P1/unknown | P1/unknown |
| Number of individual model reconstructions | 15 | 15 | 15 | 15 |
| *c*^2^, CorMap *P*-values for fit | 1.971, 0.058567 | 6.841, 4.75E-23 | 2.774, 0.058567 | 2.921, 0.000117 |
| *Atomistic modelling methods* | Comparison to Alphafold3 predicted model, ensemble optimization modelling | | | |
|  | Ct_EST | shCt_EST | Mt_EST | shMt_EST |
| Software | CRYSOL (Svergun *et al.*, 1995) | | | |
| *q-*range for fit (*q_min_* – *q_max_*; Å^-1^, nm^-1^) | 0.0096-0.4748 | 0.0068-0.4748 | 0.0068-0.4748 | 0.0096-0.4748 |
| *c*^2^, CorMap *P*-values for fit | 3.900, 1.75e-09 | 28.171, 1.54e-40 | 20.716, 5.76e-46 | 8.801, 4.13e-35 |
| Software | EOM 3.0, ranch (ATSAS 3.2.1, r14885), GAJOE 2.1 (ATSAS 3.2.1, r14885) | | | |
| *q-*range for fit (*q_min_* – *q_max_*; Å^-1^, nm^-1^) | 0.0096-0.4748 | - | 0.0068-0.4748 | - |
| Number of models in pool | 10000 | - | 10000 | - |
| Input assignment | 1-28: Disordered  29-216: Structure from Alphafold3  217-229: Disordered |  | 1-29: Disordered  30-213: Structure from Alphafold3 |  |
| Pool average Rg (Å) | 21.20 | - | 21.09 | - |
| Pool histogram Rg (Å) | 21.14 |  | 21.02 |  |
| Ensemble average Rg (Å) | 22.10 |  | 21.31 |  |
| Ensemble histogram Rg (Å) | 22.04 |  | 21.24 |  |
| Pool average Dmax (Å) | 78.80 |  | 77.19 |  |
| Pool histogram Dmax (Å) | 78.16 |  | 76.50 |  |
| Ensemble average Dmax (Å) | 78.74 |  | 70.23 |  |
| Ensemble histogram Dmax (Å) | 78.03 |  | 69.64 |  |
| Pool average Ca(N)-Ca(C) distance (Å) | 53.26 |  | 43.09 |  |
| Pool histogram Ca(N)-Ca(C) distance (Å) | 52.14 |  | 42.22 |  |
| Ensemble average Ca(N)-Ca(C) distance (Å) | 60.94 |  | 53.70 |  |
| Ensemble histogram Ca(N)-Ca(C) distance (Å) | 59.89 |  | 53.16 |  |
| Rflex – ensemble (%) | 44.93 |  | 44.68 |  |
| Rflex – random pool (%) | 85.01 |  | 86.86 |  |
| Rsigma | 0.36 |  | 0.25 |  |
| Number of selected models | 3 |  | 3 |  |
| Model names | Ct1, Ct2, Ct3 |  | Mt1, Mt2, Mt3 |  |
| Model Rg (Å) | 23.50, 21.70, 22.10 |  | 21.40, 20.70, 21.40 |  |
| Model Dmax (Å) | 93.20, 79.00, 74.20 |  | 67.30, 65.10, 71.90 |  |
| Model Fraction of ensemble | 0.09, 0.18, 0.73 |  | 0.44, 0.11, 0.44 |  |
| *c*^2^, CorMap *P*-values for ensemble fit | 2.420, 1.45e-05 | - | 6.890, 7.63e-22 | - |
| SASBDB accession codes | SASDXQ6 | SASDXR6 | SASDXS6 | SASDXT6 |

**
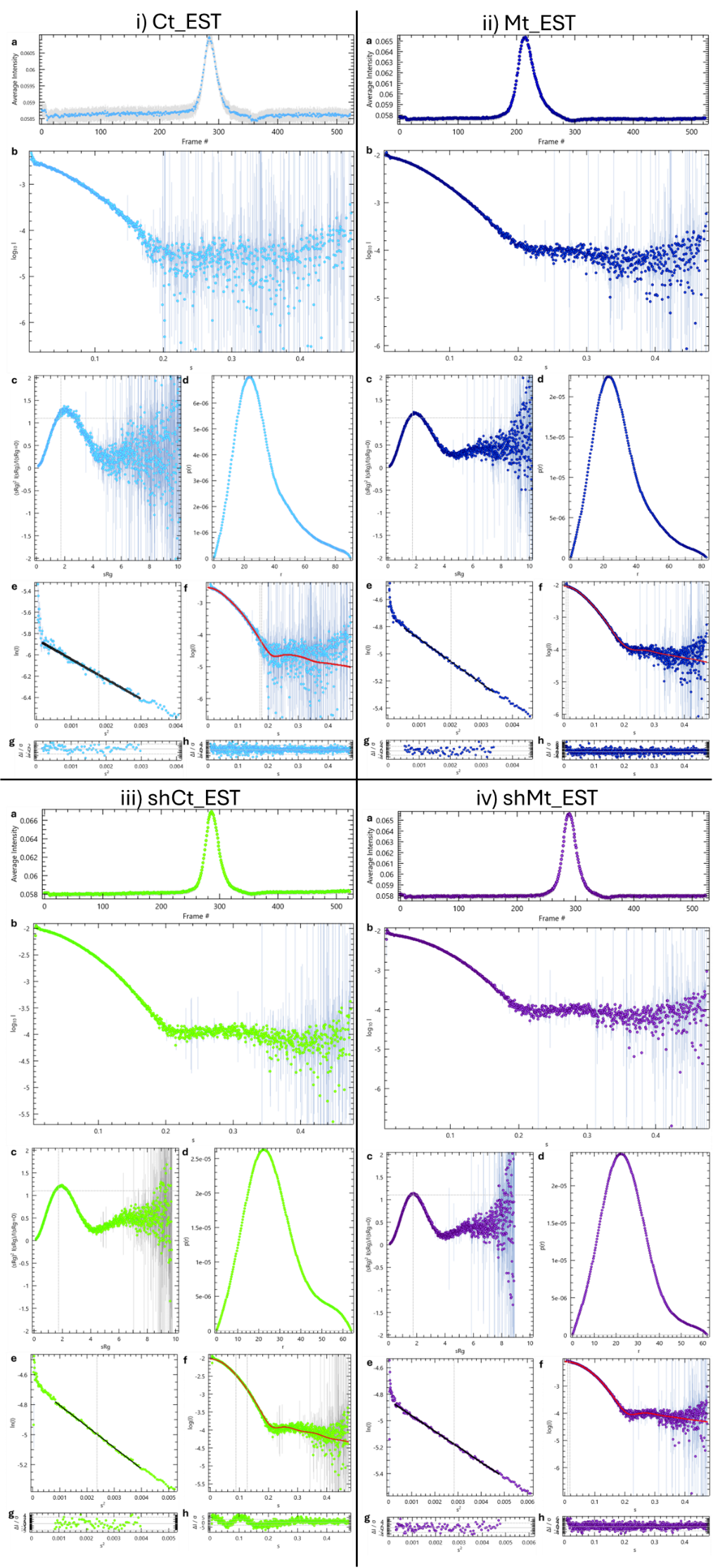
**

**SI Figure 8:** BioSAXS results for i) Ct_EST (cyan), ii) Mt_EST (blue), iii) shCt_EST (green), and iv) shMt_EST (purple). The figures for each protein are: a) SEC scattering profile, b) log linear plot, c) Dimensionless Kratky plot, d) P(r) plot, e) Guinier plot with gradient indicated on a black line of best fit. f) fit of P(r) to experimental data, g) residuals for Guinier plot, h) residuals for P(r) fit.

**
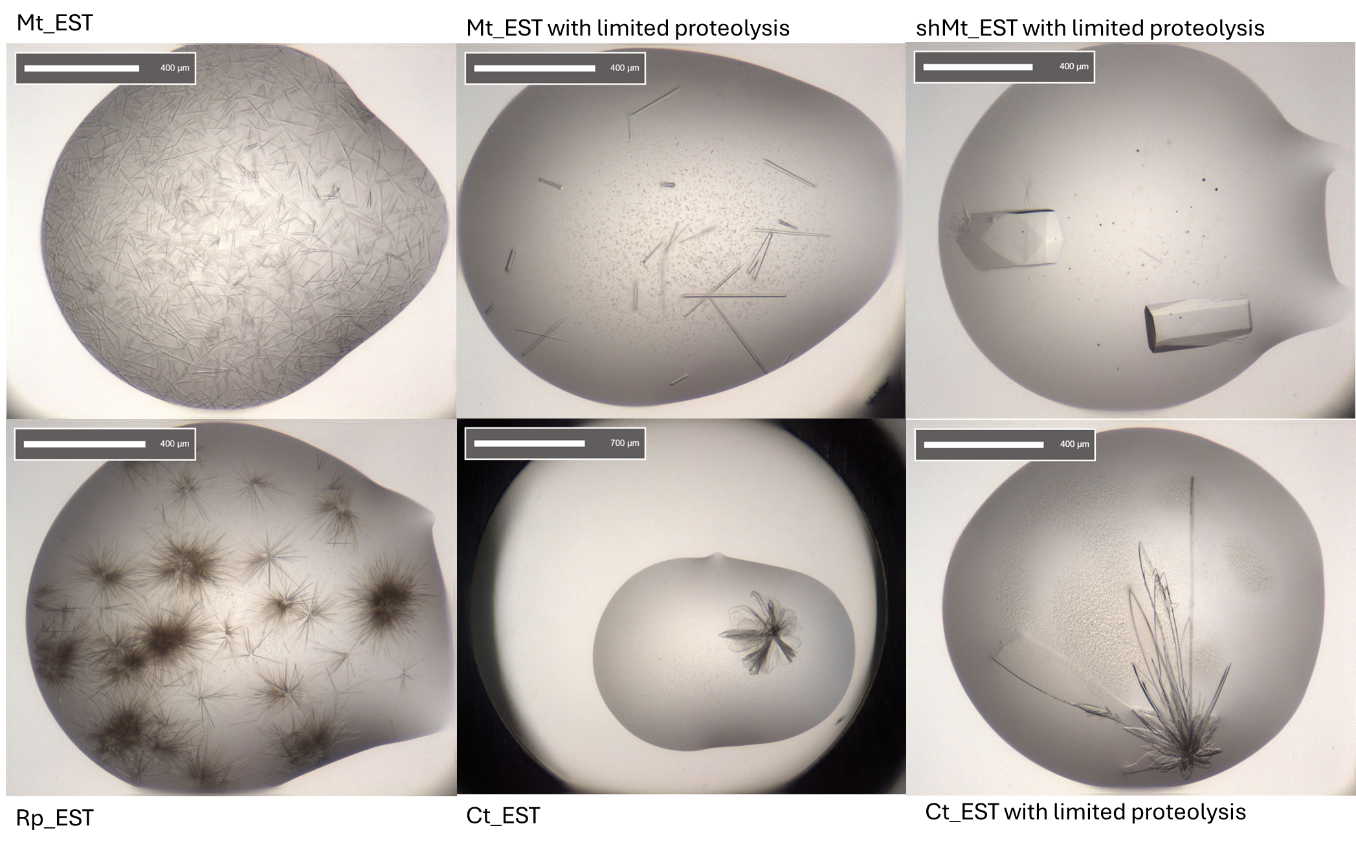
**

**SI Figure 9.** Crystallisation of Rp_EST, Ct_EST and Mt_EST.

**SI Table 5.** Crystallography statistics for shCt_EST

|  | shCt_EST |
| --- | --- |
| PDB ID | 9OTV |
| **Data Collection** |  |
| Space Group | P1 |
| Cell Dimensions |  |
| *a, b, c* (Å) | 37.7 51.8 56.4 |
| α, β, γ (^o^) | 86.7, 73.3, 90.0 |
| Resolution (Å) | 53.95 - 1.90 (1.93 - 1.90) |
| R_meas_ | 0.161 (1.020) |
| R_pim_ | 0.083 (0.526) |
| I/σI | 4.6 (1.2) |
| CC_1/2_ | 0.993 (0.704) |
| Completeness (%) | 95.3 (94.0) |
| Multiplicity | 3.7 (3.6) |
|  |  |
| **Refinement** |  |
| Resolution (Å) | 53.95 - 1.90 |
| No. of unique reflections | 30718 (2781) |
| R_work_/R_free_ | 0.2388/0.2642 |
| No. of atoms | 3043 |
| B-factors (Å^2^) |  |
| Protein | 20.11 |
| Ligand | 28.34 |
| Water | 28.59 |
| RMSD bond lengths (Å) | 0.091 |
| RMSD bond angles (^o^) | 1.21 |
| Ramachandran favored/outliers (%) | 97.53/0.0 |
| Clashscore | 1.07 |

**
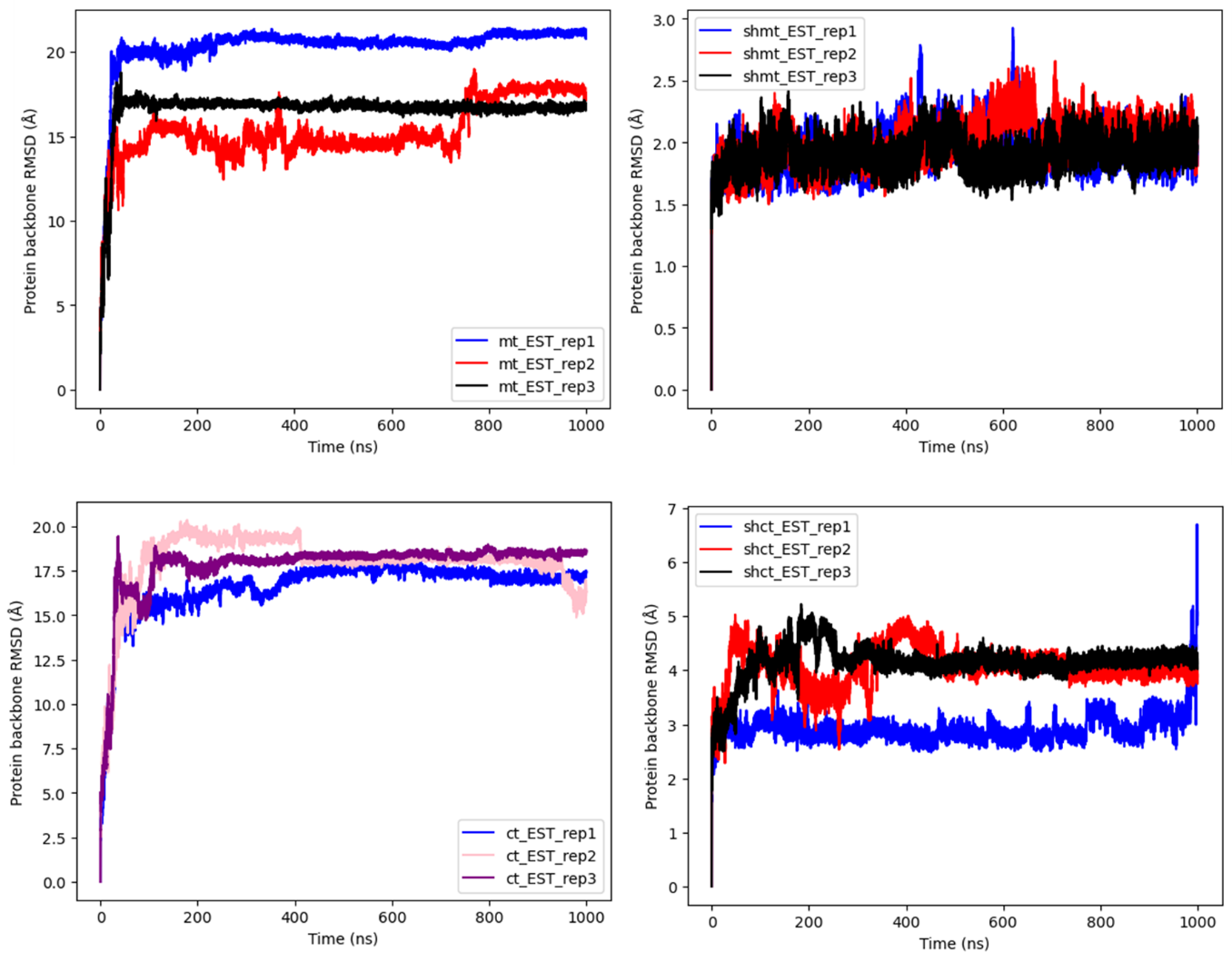
**

**SI Figure 10. A.** Stability of the protein backbone during the molecular dynamics (MD) simulation of Ct_EST, shCt_EST, Mt_EST and shMt_EST for n=3 independent simulation runs.

**SI Table 6.** Evaluating RMSD cut-offs for hierarchical clustering of N-terminal conformations of Mt_EST and Ct_EST.

|  | **Cutoff (****Å)** | **Number of Clusters** | **Mean Average Distance (Å)** | **Maximum Average Distance (Å)** | **Mean Standard deviation within clusters (Å)** | **Average Cluster Distance (Å)** |
| --- | --- | --- | --- | --- | --- | --- |
| Mt_EST | 2 | 605 | 1.29 | 1.97 | 0.17 | 5.7 |
|  | 3 | 76 | 2.1 | 2.69 | 0.38 | 6.05 |
|  | 4 | 19 | 2.73 | 3.38 | 0.6 | 6.46 |
|  | 5 | 8 | 3.28 | 4.1 | 0.85 | 6.74 |
|  | 6 | 5 | 3.58 | 4.76 | 1.06 | 6.92 |
| Ct_EST | 2 | 664 | 1.37 | 2 | 0.16 | 7.35 |
|  | 3 | 49 | 2.24 | 2.89 | 0.43 | 7.01 |
|  | 4 | 13 | 2.88 | 3.32 | 0.7 | 7.42 |
|  | 5 | 5 | 3.44 | 3.86 | 0.91 | 8.17 |
|  | 6 | 2 | 3.85 | 3.96 | 1.2 | 10.01 |


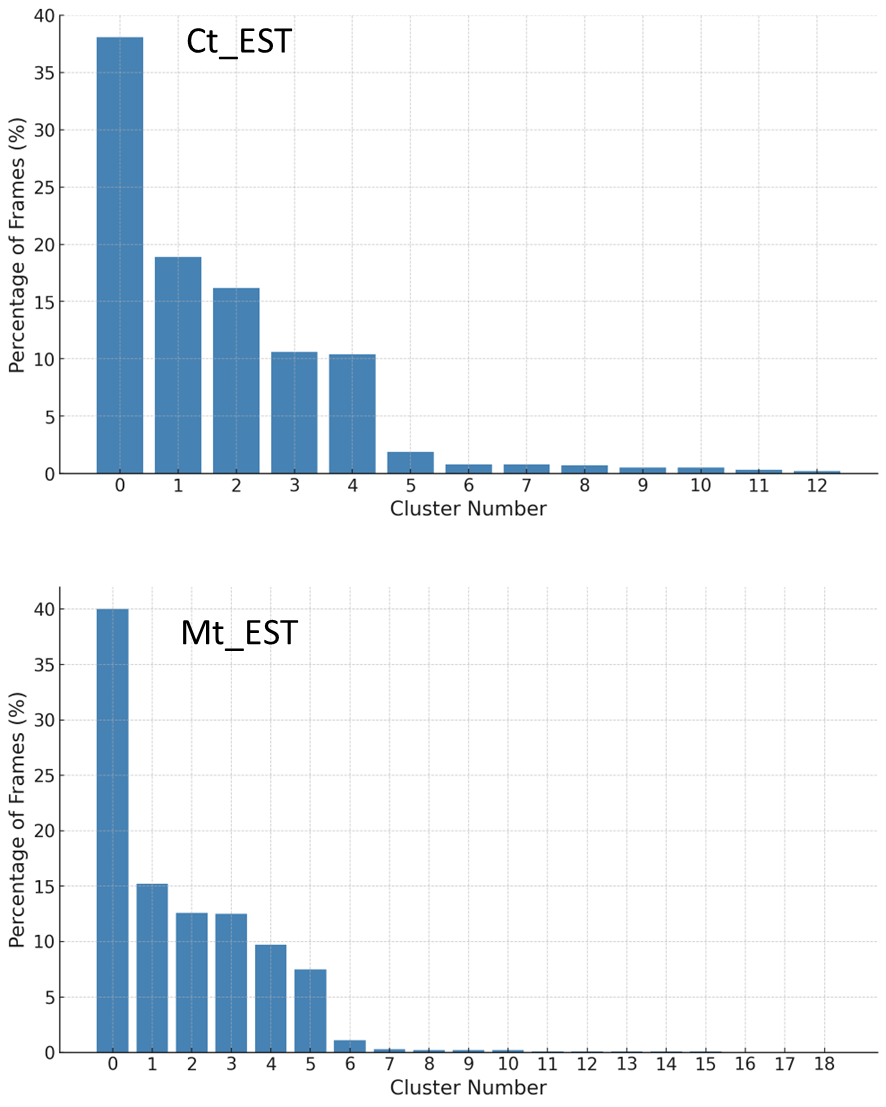


**SI Figure 11**. Cluster populations at an RMSD cut-off of 4 Å.

**
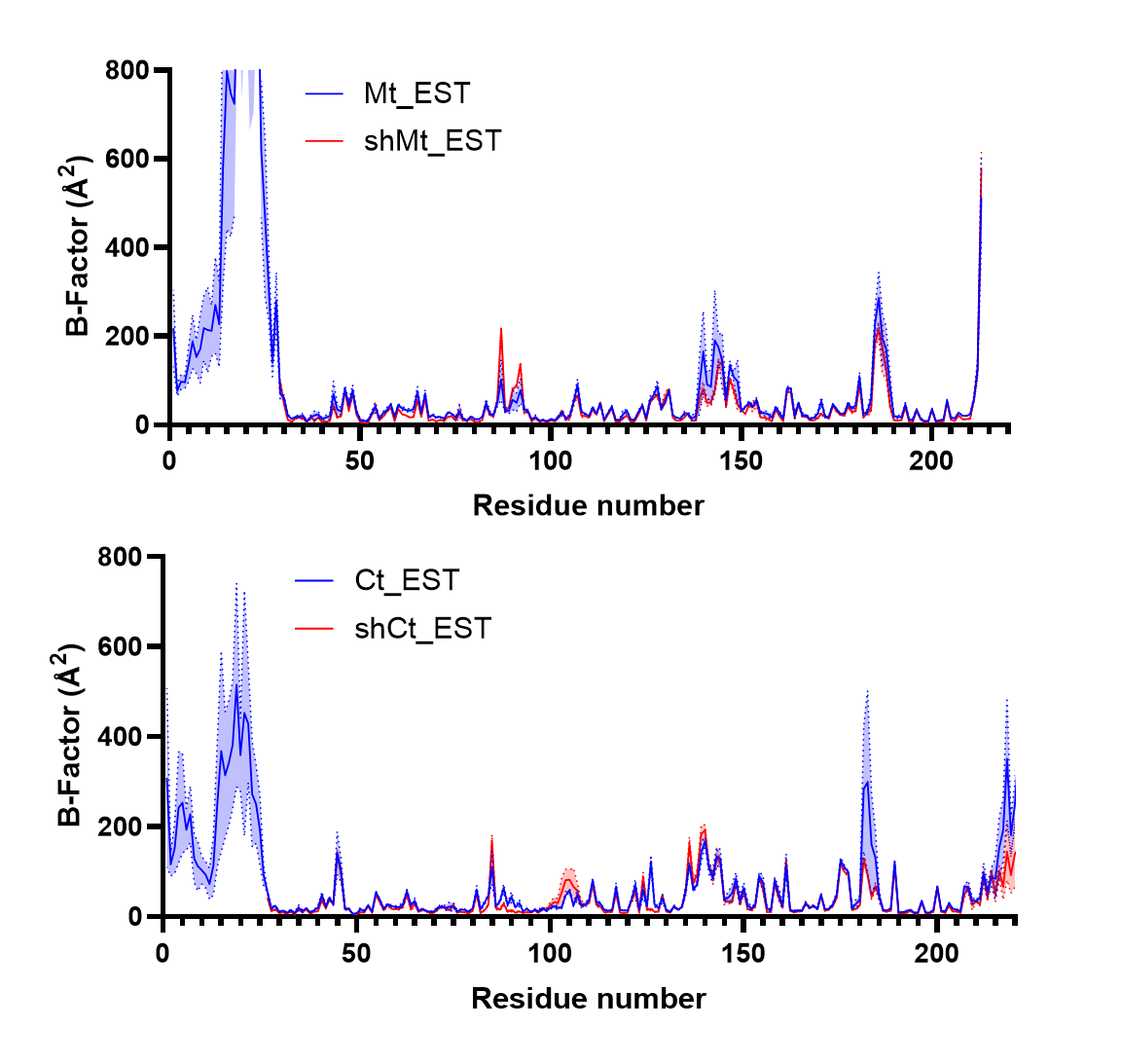
**

**SI Figure 12.** The per residue dynamics of the full-length (Ct-EST and Mt_EST) compared with their N-terminus truncated proteins (shCT_EST and shMt_EST, where the residue numbers for the shorter proteins are such that they are aligned with the full-length versions. The solid lines represent the mean from replicate simulations (n=3) and the transparent shaded regions of the same colour bound by the dashed lines represents the standard error from the mean (SEM).

**
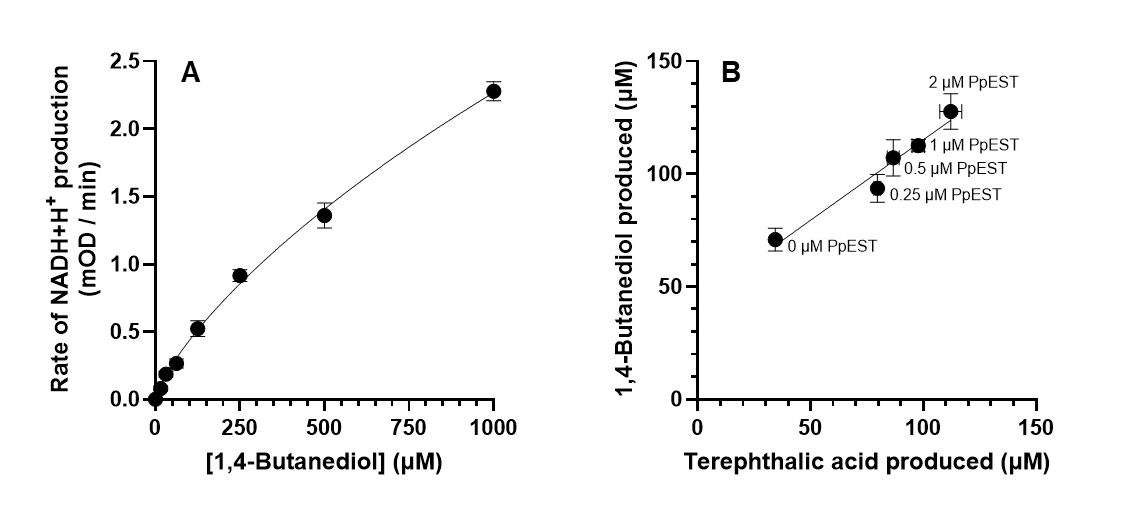
**

**SI Figure 13:** Equine alcohol dehydrogenase (EqAD) assay for detecting 1,4-butandiol and derived products from PBAT and PBSA degradation. **A.** Concentration dependent increase of 1,4-butanediol dehydrogenation when measuring absorbance at 340 nm to detect NADH + H^+^ formation. Assays contained 0.1 U/ml of EqAD and 250 μM NAD in 50 mM Tris pH 8. **B.** Comparison of the PBAT degradation products produced by increasing concentrations of PpEST when measuring 1,4-butandiol and derived products with the EqAD assay compared to terephthalic acid and derived products detected by measuring absorbance at 250 nm.

**SI Figure 14.** *In vitro* pNP-butyrate hydrolysis activity of AlinE4, ppEST, Ct_EST, shCt_EST, Mt_EST, shMt_EST, Rp_EST and shRp_EST. Assays were performed at room temperature in 50 mM tris pH 8.0, with 0.05-0.1 μM of the respective enzyme (n = 3). Error bars represent the standard error from the mean.

**References**

1. Aragão D, Aishima J, Cherukuvada H, Clarken R, Clift M, Cowieson NP, Ericsson DJ, Gee CL, Macedo S, Mudie N, et al. (2018) MX2: a high-flux undulator microfocus beamline serving both the chemical and macromolecular crystallography communities at the Australian Synchrotron. J Synchrotron Radiat 25:885–891.

2. Kabsch W (2010) XDS. Acta Crystallogr D Struct Biol 66:125–132.

3. Evans PR, Murshudov GN (2013) How good are my data and what is the resolution? urn:issn:0907-4449 69:1204–1214.

4. Vonrhein C, Flensburg C, Keller P, Sharff A, Smart O, Paciorek W, Womack T, Bricogne G (2011) Data processing and analysis with the autoPROC toolbox. Acta Crystallogr D Struct Biol 67:293–302.

5. McCoy AJ, Grosse-Kunstleve RW, Adams PD, Winn MD, Storoni LC, Read RJ (2007) Phaser crystallographic software. J Appl Crystallogr 40:658–674.

6. Abramson J, Adler J, Dunger J, Evans R, Green T, Pritzel A, Ronneberger O, Willmore L, Ballard AJ, Bambrick J, et al. (2024) Accurate structure prediction of biomolecular interactions with AlphaFold 3. Nature 2024 630:8016 630:493–500.

7. Jumper J, Evans R, Pritzel A, Green T, Figurnov M, Ronneberger O, Tunyasuvunakool K, Bates R, Žídek A, Potapenko A, et al. (2021) Highly accurate protein structure prediction with AlphaFold. Nature 2021 596:7873 596:583–589.

8. Bricogne G, Blanc E, Brandl M, Flensburg C, Keller P, Paciorek W, Roversi P, Sharff A, Smart OS, Vonrhein C, et al. (2017) BUSTER.

9. Ishida T, Kinoshita K (2007) PrDOS: prediction of disordered protein regions from amino acid sequence. Nucleic Acids Res 35:W460–W464.

10. Lotthammer JM, Ginell GM, Griffith D, Emenecker RJ, Holehouse AS (2024) Direct prediction of intrinsically disordered protein conformational properties from sequence. Nature Methods 2024 21:3 21:465–476.
